# Prognostic factors associated with changes in knee pain outcomes, identified from initial primary care consultation data. A systematic literature review

**DOI:** 10.1080/07853890.2023.2165706

**Published:** 2023-01-27

**Authors:** Thomas S. Collier, Tom Hughes, Rachel Chester, Michael J. Callaghan, James Selfe

**Affiliations:** a Pure Physiotherapy Specialist Clinics, Norwich, UK; b School of Health Sciences, Faculty of Medicine and Health, University of East Anglia, Norwich Research Park, Norwich, UK; c Department of Health Professions, Manchester Metropolitan University, Manchester, UK; d Football Medicine and Science Department, Manchester United Football Club, Manchester United Training Centre, Manchester, UK; e Manchester University NHS Foundation Trust, Manchester, UK

**Keywords:** Primary care, prognosis, knee pain, prognostic factors, musculoskeletal, systematic literature review

## Abstract

**Background:**

Data collected during initial primary care consultations could be a source of baseline prognostic factors associated with changes in outcome measures for patients with knee pain.

**Objectives:**

To identify, appraise and synthesize studies investigating prognostic factors associated with changes in outcome for people presenting with knee pain in primary care.

**Methods:**

EMBASE, CINAHL, AMED, MEDLINE and MedRxiv electronic databases were searched from inception to March 2021 and repeated in August 2022. Prospective cohort studies of adult participants with musculoskeletal knee pain assessing the association between putative prognostic factors and outcomes in primary care were included. The Quality in Prognostic Studies (QUIPS) tool and The Modified Grading of Recommendations Assessment, Development and Evaluation (GRADE) framework, specific to prognostic reviews were used to appraise and synthesize the evidence respectively.

**Results:**

Eight studies were included. Eight knee pain outcomes were identified. Methodological and statistical heterogeneity resulted in qualitative analysis. All evidence was judged to be of low to very low quality. Bilateral knee pain (multivariable odds ratio (OR) range 2.60–2.74; 95%CI range 0.90–8.10, *p* value = 0.09) and a lower educational level (multivariable (OR) range 1.74–5.6; 95%CI range 1.16–16.20, *p* value = <0.001) were synonymously associated with persisting knee pain at 12-month follow up. A total of 37 univariable and 63 multivariable prognostic factors were statistically associated with outcomes (*p* ≤ 0.05) in single studies.

**Conclusions:**

There was consensus from two independent studies that bilateral knee pain and lower educational level were associated with persistent knee pain. Many baseline factors were associated with outcome in individual studies but not consistently between studies. The current understanding, accuracy and reliability of the prognostic value of initial primary care consultation data for knee pain outcomes are limited. This review will provide an essential guide for candidate variable selection in future primary care prognostic confirmatory studies.

**Key messages**
- Bilateral knee pain and lower educational level were associated with persistent knee pain.
- Many baseline factors were associated with outcome in individual studies but not consistently between studies.
- The current understanding, accuracy and reliability of the prognostic value of initial primary care consultation data for knee pain outcomes are limited.

## Introduction

Musculoskeletal (MSK) pain is a leading cause of disability worldwide and is likely to rise globally with an ever-growing population and increased life expectancy [1,2]. MSK pain accounts for 22% of the total burden of ill health in the UK [3]. Knee pain is one of the most common complaints observed, with prevalence rates in the general population estimated to be between 19 and 35% [4,5].

People suffering from knee pain are frequently managed in primary care and represent approximately 10% of all primary care consultations for MSK conditions [6]. For the purposes of this review, primary care refers to services provided by registered medical or healthcare practitioners (generally in community settings), which provide patients with an initial point of contact or consultation where they can seek advice or assessment of a health complaint or condition. Examples include general practitioners, paramedic practitioners, physician associates, first contact physiotherapy practitioners and nurse practitioners.

During initial consultations, practitioners typically conduct a detailed review of the history of the current condition and perform a clinical assessment to establish a working diagnosis. Current primary care management models recommend an array of further diagnostic investigations or management options; this can include advice, physiotherapy, pharmacological management or onward specialist referral (i.e. transfer to secondary care) [7–9]. However, selecting the most appropriate course of action can be challenging and clinical decisions are usually influenced by, and can be biased towards, a practitioner’s scientific knowledge and skillset [10].

To assist practitioners, evidence can be considered from prognostic factor research [11]. Prognostic factors are any measurements, characteristics or variables (such as routine data collected during initial consultations, for example) that are associated with a change in risk or probability of the occurrence of a future health-related outcome among patients with a defined health condition [12–16]. Variation in the values, levels or categories of individual factors will result in risk or probability differences for the occurrence of health outcomes between patients [11]. This means that prognostic factors are useful to explain why some patients have a better or worse prognosis than others [14]. Furthermore, identification of prognostic factors can inform treatment recommendations and help facilitate development of innovative treatment approaches if there is evidence of a causal link between the factor and outcome [14].

Multiple prognostic factors can also be used in combination to develop clinical prediction models, providing patients with individualized estimates of risk or probability of a future health outcome at the point of consultation [17]. Prognostic models can also facilitate stratified management, where bespoke clinical management decisions can be informed by an individual’s risk or probability estimate and profile of prognostic factors [16]. Therefore, if robust prognostic factors for the likely course of knee pain could be identified at initial consultation, this may improve the effectiveness and efficiency of various clinical decisions, thus benefitting patients and health care providers alike.

Previous studies conducted in secondary care settings (i.e. acute hospitals) have identified a number of prognostic factors associated with worsening knee pain outcomes in adults, including increasing age and body mass, as well as a history of sustaining a previous knee injury [18,19]. Several generic prognostic factors for MSK conditions have been established in the primary care setting such as pain intensity, widespread pain, high functional disability, somatization and movement restriction [20]. However, there is currently limited evidence related to prognostic factors associated with changes in health outcomes for people specifically suffering from knee pain.

Consequently, because of the burden of knee pain on primary care services and the potential benefits of utilizing prognostic factors in practice, there is a clear need to explore whether routine data obtained at the point of initial consultation has prognostic value. Therefore, the aim of this systematic review is to summarize, appraise and synthesize the evidence to identify prognostic factors associated with changes in knee pain outcome in adult patients, obtained from data derived from initial primary care consultations. This, to the best of our knowledge, has not been conducted previously.

### Methods

Our methodology was specified a priori and registered with the International Prospective Register of Systematic Reviews (PROSPERO) registration ID; CRD42021229699. This review was reported in accordance with the Preferred Reporting Items for Systematic Reviews and Meta-Analysis (PRISMA) guidelines [20]. Ethical approval and consent was not required in the absence of human participants.

### Data sources and search strategy

The EMBASE, CINAHL, AMED, MEDLINE and MedRxiv electronic databases were searched from inception to March 2021 and repeated in August 2022. The search strategy is presented in supplementary files 1–4. Searches were limited to original research articles published in the English language. Systematic reviews, editorials and conference abstracts were excluded. A hand search from all included articles was also undertaken to avoid omitting potentially relevant articles.

### Eligibility criteria

#### Participants

Studies were included if participants: (1) were adults aged 18 years or over; (2) sought an initial primary care consultation with a registered health care or medical professional for MSK knee pain of any duration; (3) had not received any prior management. Studies were excluded if participants: (1) underwent surgery or enrolled in post-operative knee rehabilitation; (2) had non- MSK knee pain (e.g. malignancy); (3) had referred pain from other sources (e.g. radiculopathy); (4) had systemic inflammatory conditions with associated knee pain (i.e. that manifested as monoarticular or polyarticular inflammatory arthropathies; (5) had a subluxation, dislocation or fracture/s; (6) had a serious lumbar pathology; (7) had been referred to secondary care management.

##### Study design

Studies were included if they: (1) were a prospective or retrospective cohort, case-control or nested case-control design; (2) specifically investigated the association between candidate prognostic factors, measured within 2 weeks of the initial primary care consultation date and outcome measures relevant to knee pain; (3) conducted multivariable analyses to adjust for the prognostic effect of other important candidate prognostic factors, such as age and biological sex. Studies were excluded if they: (1) were of any other design (*N* = 4) or; (2) were not exclusively primary care based (*N* = 29) or; (3) surgery used as an intervention (*N* = 7).

##### Prognostic factors

Studies were included if any of the following data (obtained from initial consultations) were investigated as candidate prognostic factors: (1) patient characteristics; (2) demographics; (3) recreational activities; (4) radiographic imaging; (5) blood tests; (6) knee symptoms; (7) clinical examination; (8) general health; (9) clinical or radiographic findings that are reported within 2 weeks from initial consultation. Additionally, if there was evidence of the conduct of multivariable analyses to adjust for the prognostic effects of other important prognostic factors, including age and biological sex.

##### Outcome measures

Studies were included if they investigated specific outcome measures for knee pain, in the domains of pain, function, disability, general health and quality of life scores.

### Study selection

Studies were initially screened using the title and abstract for potential full-text review by the primary author (TC). All potentially eligible full-text studies were jointly reviewed in an independent blinded manner by the primary (TC) and secondary author (TH) against all pre-defined eligibility criteria. Disagreements were resolved by discussion between the primary and secondary authors until mutual agreement was reached, no arbitration was required.

#### Data extraction

Data were extracted by one reviewer (TC) according to the Checklist for Critical Appraisal and Data Extraction for Systematic Reviews of Prediction Modelling Studies - Prognostic Factors [11] (Supplementary file 5). Extracted data were checked for consistency by all reviewers in an unblinded manner.

#### Risk of bias

Risk of bias (RoB) was assessed for all included studies using The Quality in Prognostic Studies (QUIPS) tool, by two reviewers (TC, TH) in an independent blinded manner. The QUIPS tool is a reliable method of RoB evaluation for studies of prognostic factors through six independent domains, which include: (1) study participation; (2) study attrition; (3) prognostic factor measurement; (4) outcome measurement; (5) study confounding; (6) statistical analysis and reporting [21]. Studies were classified as low, moderate, or high RoB based on the QUIPS tool guidance for ROB judgements (see Supplementary file 6) [21,22]. Any disagreements were resolved through discussions. A third reviewer, acting as an arbitrator, was not required.

#### Data analysis and synthesis

Extracted data and QUIPS appraisals were tabulated for each included study to facilitate the evidence synthesis and assess study heterogeneity (Table 1). Data synthesis was conducted according to the modified Grading of Recommendations Assessment, Development and Evaluation (GRADE) framework to assess and grade the quality of evidence [23]. All statistically significant prognostic factors that were investigated by single studies or those that were investigated by two or more studies were tabulated and grouped according to each knee outcome (Table 2). Where homogenous effect measures were reported for the same prognostic factor across two or more studies, these were summarized using forest plots (Figures 2 and 3). Key judgements for each prognostic factor in the following modified GRADE domains were made: (1) study limitations; (2) consistency of results; (3) effect sizes; (4) precision of results; (5) publication bias; and (6) overall quality (Supplementary files 7 and 8). Decisions on prognostic value were made when considering effect size, subjective interpretation of 95% confidence interval (CI) width and *p* value size. Effect estimates were deemed to be of potential significant prognostic value if there was evidence of a moderate effect size (in the absence of excessively wide CIs) or small effect size (with narrow CIs) and were displayed in bold text and underlined (Table 2). Thresholds for odds ratio (OR) and hazard ratio (HR) categorization were obtained and adapted from Huguet et al. [23]. If OR and HR effects were greater than 1, these were defined as small if OR or HR effect sizes were between 1 and 2.49, moderate if effect sizes were between 2.50 and 4.24, or large if effect sizes were >4.25. In the event of that OR and HR values were less than 1, effect sizes were defined as small if between 0.99 and 0.66, moderate if between 0.65 and 0.32 and large if <0.32.

**Table 1.** Characteristics and Quality in Prognosis Studies (QUIPS) assessment for included studies.

Characteristics											QUIPS score per domain					
	Participants, Setting, design and sample size	Outcomes		Prognostic factors			Missing data		Analyses		Participation	Attrition	Prognostic factors	Outcome measure	Adjustment for other prognostic factors	Statistical Reporting
	Description and study deign	Type and Number of outcomes	Duration of FU (months)	Number and type of prognostic factors	Timing of prognostic factor and outcome measurement	Handling of prognostic factors in the analysis	Number of participants missing data (%)	Handling of missing data	Modelling method	Sample size calculation						
[24]	Practitioners: Not stated Participants: males (*N* = 134 and female (*N* = 571) recruited from the Cohort Hip and Cohort Knee study in the Netherlands. Design: Prospective Cohort Sample size: *N* = 705	Pain (NRS)	40	Demographics: Age, gender, BMI, ethnicity Health: medication, alcohol use, smoking history, vitamin or supplement use Medical history: asthma, chronic sinusitis, CVD, hypertension, gastric ulcer, gallstones, liver disease, diabetes, thyroid gland disease, epilepsy, cancer, severe skin disease, other MSK disease. Knee symptoms and signs: WOMAC pain, stiffness and physical functioning Physical examination: Pain in ipsilateral hip, morning stiffness <30 min, palpable warmth, joint space tenderness, bony enlargement, crepitus, positive refill test, pain during knee ROM, pain during hip ROM, Bouchard swelling, Heberden node knee pain, range of motion Radiographs: knee and hip Blood tests: ESR Psychosocial: Pain coping behaviour, education level	Prognostic Factors: Baseline Outcome measure: Every year up to 5 years	Not stated	38 (5)	Not specified	Univariate multinomial regression analyses followed by latent class growth analysis	No	Low	High	High	Low	High	High
[25]	Practitioners: 40 general practitioners Participants: males (*N* = 211) and females (*N* = 269) recruited from five municipalities in the Netherlands. Design: Prospective Cohort Sample size: *N* = 480	Persisting knee symptoms (Dichotomized into recovered symptoms and persistent symptoms)	12	Demographics: mean age, age >60 years, women, mean BMI, BMI >25 Health: Presence of comorbidity in skeletal system, presence of other comorbidities Patient characteristics: private insurance, paid employment >8 h/week, sport participation Knee symptoms and signs: pain level, duration of symptoms, history of non-traumatic knee symptoms, history of traumatic knee symptoms, presence of locked knee, bilateral symptoms, recurrent symptoms feeling of giving way, limited when walking stairs, WOMAC pain and stiffness and physical functioning Physical examination: warm, swollen, crepitus PROM, crepitus AROM, varus/ valgus alignment, PROM pain, AROM pain, anterior drawer, patella alignment assessment, joint swelling, pain on internal hip rotation, restriction of internal hip rotation, presence of: Heberden’s nodes; Bakers cysts; prepatellar bursitis, ITB pain. Psychosocial: education level, mean Kinesiophobia score, Kinesiophobia score >25′	Prognostic Factors: Baseline Outcome measure: 1 year except disability and pain; every 3 months over 1 year	Dichotomization of candidate factors with continuous data	69 (14)	Multiple imputation	Univariable logistic regression followed by multivariable logistic regression with backward variable selection	No	Low	Low	Mod	Mod	Mod	Mod
[26]	Practitioners: 40 general practitioners Participants: males (*N* = 277) and females (*N* = 272) were recruited from the research network HONEUR (part of a prospective observation cohort) in the Netherlands. Design: Prospective Cohort Sample size: *N* = 549	Unfavourable outcome (categorized as persistent knee symptoms at 6-year FU or having undergone knee replacement surgery during FU).	3,6,9,12 and 54	Demographics: age, sex, and comorbidity Health: Health-related quality of life, advice given by the GP, knee medication, visits to health care professionals and operations Activity and sports participation: Daily activities and physical exercise Knee symptoms and signs: History of previous knee injuries or operations, duration and recurrence of knee symptoms, knee pain level, WOMAC pain stiffness, and function domains. Physical examination: Knee alignment, joint effusion, palpation, temperature, collateral ligaments, a joint line tenderness, assessment of effusion, passive knee ROM in flexion and extension, meniscal tests and knee stability tests. Psychosocial: Composition of household, education level, coping, sick leave from daily activities and data on impact of the knee symptom as a hindrance in terms of daily activities.	Prognostic Factors: Baseline Outcome measures: 3,6,9,12 and 54 months	Dichotomization of candidate factors with continuous data	209 (38)	Multiple imputation	Univariable logistic regression followed by multivariable logistic regression with backward variable selection	No	Mod	High	Mod	Low	High	High
[27]	Practitioners: 40 General Practitioners Participants: Adolescents and adults with traumatic knee symptoms from general practice. Males (*N* = 90) and females (*N* = 82) were recruited from the research network HONEUR (part of a prospective observation cohort) in the Netherlands. Design: Prospective cohort Sample size: *N* = 172	Persistent knee symptoms (dichotomized into recovered symptoms and persistent symptoms)	3,6,9,12 and 54	Demographics: Age, gender, BMI >25 Health: Comorbidities of the skeletal system, other non-skeletal comorbidities, self-rated poor health, Sports: Sports hindrance and level of daily activities. Knee symptoms and signs: Duration >3 months, recurrent symptoms, bilateral symptoms, pain level, self-reported warmth of knee, self-reported knee swelling, self-reported knee crepitus, knee locking, knee instability, history of knee symptoms, WOMAC pain, stiffness and function domains Psychosocial: Education level, sick leave	Prognostic Factors: Baseline Outcome measures: 12 and 54 months	Dichotomization of candidate factors with continuous data	72 (41)	Multiple imputation	Univariable logistic regression followed by multivariable backward logistic regression	No	Mod	Mod	High	Mod	Mod	Mod
[28]	Practitioners: 40 General Practitioners Participants: Males (*N* = 185) and females (*N* = 143) were recruited from the research network HONEUR (part of a prospective observation cohort) in the Netherlands. Design: Prospective cohort Sample size: *N* = 328	Self-reported perceived recovery (dichotomized into recovered symptoms and persistent symptoms)	3,6,9,12 and	Demographics: Age, gender, BMI Health: Co-morbidities of skeletal system, other non-skeletal comorbidities, mental health, general health. Patient characteristics: employment type, sport participation. Knee symptoms and signs: injury history including trauma during sport, fall on the knee, weight bearing on knee, rotational trauma, foot/leg blocked, immediate pain at trauma, immediate effusion after trauma, popping sensation at trauma, whether continuation of activity was possible, past history of traumatic knee symptoms, history of non-traumatic knee symptoms,; symptoms at time of consultation including pain level, self-reported knee warmth, self-reported knee swelling, self-reported knee crepitus, locking of the knee, knee instability, limitations on work/study/ daily function. Physical examination: Patella alignment, pain during AROM flexion, pain during AROM extension, pain during PROM flexion, pain during PROM extension, laxity during valgus stress test 30 degrees, laxity during varus stress test 30 degrees, laxity during anterior drawer test, effusion of popliteal fossa, McMurray meniscal test, Apley grinding test, Apley traction test Psychosocial: Education level	Prognostic Factors: Baseline Outcome measures: 3, 6, 9, 12 and 54 months	Not stated	32 (10) lost at 1 year. 145 (44) lost at 6 years	Multiple imputation	Univariable logistic regression followed by backward logistic regression	No	Mod	Low	High	Mod	Mod	Mod
[29]	Practitioners: Not stated Participants: males (*N* = 283) and females (*N* = 338) were recruited from three practices from the North Staffordshire Primary Care Research Consortium, UK. Design: Prospective cohort Sample Size: N = 766	Functional outcome using WOMAC-PF scores.	18	Demographics: Age, gender Health: Alcohol consumption, smoking status, self-rated health, number of selected comorbid health conditions Patient characteristics: Occupation, marital status Knee symptoms and signs: Pain location, pain level, chronic pain grade, whole leg pain, widespread pain, number of days in pain (last 6 months) and pain episode duration. Psychosocial: Anxiety, depression, sociodemographic characteristics social network index	Prognostic Factors: Baseline Outcome measures: 18 months	Not stated	198 (26)	Not stated	Univariable cox regression followed by backwards stepwise cox regression with backwards variable selection	No	Low	High	Mod	Low	High	High
[30]	Practitioners: Not stated Participants: Three practices from the North Staffordshire Primary Care Research Consortium in the UK. Design: Prospective cohort Sample size: *N* = 621	Functional outcome using WOMAC-PF scores.	18	Demographics: Age, BMI Knee symptoms and signs: Knee pain severity, first-degree relative with arthritis, previous meniscectomy, contralateral total knee replacement, bilateral knee pain, duration of morning stiffness, inactivity gelling, self-reported swelling in past month, incident knee pain, giving way and locking. Physical examination: Clinical osteoarthritis of the hand, observed intermalleolar gap in standing, observed intercondylar gap in standing, severity of knee joint effusion, fixed flexion deformity of the knee, pain provocation on PFJ compression, observed bony enlargement, local tender point count, anteroposterior instability mediolateral instability, range of hip internal rotation, range of knee flexion, maximal isometric knee extensor strength, maximal isometric knee flexor strength, palpation of crepitus, timed single leg standing balance, hip rotation PROM, knee flexion PROM Radiography: Severity of knee changes Psychosocial: Anxiety	Prognostic Factors: Baseline Outcome measures: 18 months	Not stated	124 (19)	Not stated	Univariate cox regression followed by multivariate cox regression with backwards variable selection	No	Low	High	Mod	Low	High	High
[31]	Practitioners: 49 General Practitioners Participants: Males (*N* = 51) and females (*N* = 200) were recruited from 61 general practices (97 GPs) in the Netherlands. Design: Prospective cohort Sample size: *N* = 251	Perceived recovery (dichotomized into yes or no groups), Pain intensity measured as NRS. Functional outcomes measured through WOMAC	3 and 12	Demographics: Age, sex, BMI Patient characteristics: Work status, marital status, number of children (<5 years) in household Health: Smoking status, quality of life, perceived general health and vitality, comorbidities, physical activity, ACSM position stand, social support Knee symptoms and signs: Symptom duration, location, history, severity, perceived cause of the complaint, presence of menopause, use of pain medication Psychosocial: Education, pain coping strategies, distress, Kinesiophobia	Prognostic Factors: Baseline Outcome measures: 3 and 12 months	Dichotomization of candidate factors with continuous data Categorical (several coping strategies, distress, and the 2 Kinesiophobia subscales. Continuous variables: (Age, duration of the knee complaint, pain, WOMAC pain, stiffness and function, vitality and social support	3 months: 223 (92%) 12 months: 203 (80%).	Not stated	Perceived recovery: Cox regression with backward variable selection Pain and function: Univariable analysis followed by multiple linear regression using backward variable selection	No	Low	High	Mod	Mod	Mod	High

Key: Checklist for critical appraisal and data extraction for systematic reviews of prognostic factor studies (CHARMS-PF); Quality in prognosis studies (QUIPS); Follow-up (FU); body mass index (BMI); Cardiovascular disease (CVD); MSK; Range of movement (ROM); Western Ontario and McMaster Universities Osteoarthritis Index (WOMAC); Western Ontario and McMaster Universities Osteoarthritis Index Physical Functioning (WOMAC-PF); Erythrocyte sedimentation rate (ESR); Active range of movement (AROM); Passive range of movement (PROM); Iliotibial band (ITB); Activities of daily living (ADL’s); General Practitioner (GP); American College of Sports Medicine (ACSM); Patellofemoral joint (PFJ).

QUIPS score per domain: low risk of bias; moderate risk of bias; high risk of bias.

**Table 2.** Summary of synthesis of significant prognostic factors, or prognostic factors that were investigated in two or more with associated GRADE evaluation.

Summary of synthesis of prognostic factors									Adapted GRADE criteria				
Specific outcome	Number of studies	Potential prognostic factors	Authors	Effect Measure	Univariable effect size (95%CI)	*p* Value	Multivariable effect size (95%CI)	*p* Value	Consistency of results	Effect size	Precision of results	Publication bias	Overall Quality
		Three-month follow up											
Pain (NRS)	2	Sex BMI > 30 Duration of the knee complaint Cause overload during unusual activities Baseline pain PCI Distress Middle *vs.* low Coexisting MSK complaints ACSM position stand recommendations	[31] [31] [31] [31] [31] [31] [31] [31]	RC RC RC RC RC RC RC RC	0.70 (−1.41 to 0.01 0.84 (−0.13 to 1.81) −0.20 (−0.42 to 0.02) −1.65 (−2.99 to −0.32) 0.59 (0.46–0.73) 0.38 (−0.50 to 1.27) −0.73 (2.10 to 0.64) 0.73 (−0.21 to 1.67)	<0.20 <0.20 <0.20 <0.20 <0.20 <0.20 <0.20 <0.20	**−1.01 (−1.60 to 0.42)** **0.86 (0.06–1.67)** **−0.25 (−0.44 to 0.07)** **−1.09 (−2.19 to 0.02)** **0.65 (0.53–0.78)** **−1.66 (−3.06 to −0.26)** −1.20 (−2.36 to −0.03) **0.77 (−0.01 to 1.55)**	<0.001 0.004 0.01 0.05 <0.001 0.02 0.04 0.05	- - - - - - - -	Small Small Small Small Small Small Small Small	Imprecise Imprecise Imprecise Imprecise Imprecise Imprecise Imprecise Imprecise	Likely Likely Likely Likely Likely Likely Likely Likely	Very low Very low Very low Very low Very low Low Very low Very low
		12-month follow up											
		Non-traumatic knee complaint history Baseline pain present PCI distraction high *vs.* low PCI distress high *vs.* low Vitality	[31] [31] [31] [31] [31]	RC RC RC RC RC	−1.19 (−1.97 to −0.41) 0.61 (0.46–0.75) −1.09 (−2.05 to −0.14) −3.68 (−5.82 to 1.54) 0.02 (0.0–0.05)	<0.20 <0.20 <0.20 <0.20 <0.20	**−1.31 (−1.94 to −0.67)** **0.69 (0.55– 0.82)** **−1.02 (−1.80 to 0.24)** **−2.03 (−3.93 to −0.12)** 0.02 (0.00–004)	<0.001 <0.001 0.01 0.04 0.03	- - - - -	Small Small Small Small Small	Imprecise Imprecise Imprecise Imprecise Imprecise	Likely Likely Likely Likely Likely	Very low Very low Very low Low Very low
		54-month follow up											
		Higher BMI Lower level of education Greater comorbidity Higher activity limitation scores Joint space tenderness	[24] [24] [24] [24] [24]	RR RR RR RR RR	- - - - -	- <0.05 <0.05 <0.05 <0.05	**1.10 (1.01–1.20)** **0.55 (0.23–1.31)** **2.65 (1.25–6.99)** 2.03 (0.96–4.33) **1.08 (0.51–2.29)**	- - - - -	- - - - -	Small Small Moderate Small Small	Imprecise Imprecise Imprecise Imprecise Imprecise	Likely Likely Likely Likely Likely	Very low Low Low Very low Very low
		12-month follow up											
Persisting knee symptoms	2	Age >60 years Education level Kinesiophobia Comorbidity of MSK system Non-traumatic knee history symptoms Bilateral symptoms >3-month symptom duration Crepitus of PROM extension	[25] [25] [27] [25] [25] [25] [25] [27] [25] [25]	OR OR OR OR OR OR OR OR OR OR	1.56 (1.08–2.24) Figure 2 Figure 2 1.99 (1.37–2.89) 0.83 (0.58– 1.19) 5.12 (2.97– 8.81) Figure 3 Figure 3 3.04 (1.98–4.65) 1.11 (0.75–1.66)	<0.20 <0.20 0.002 <0.20 <0.20 <0.20 <0.20 0.11 <0.20 <0.20	**2.02 (1.30–3.13)** Figure 2 Figure 2 **1.85 (1.26– 2.72)** **1.50 (0.99– 2.28)** 4.30 (2.38–7.79) Figure 3 Figure 3 2.20 (0.9–5.6) 1.91 (1.01–3.63)	- - <0.001 - - - − 0.09 - -	- Yes Yes - - - Yes Yes - -	Small Small Large Small Small Moderate Moderate Moderate Small Small	Imprecise Imprecise Imprecise Imprecise Imprecise Imprecise Imprecise Imprecise Imprecise Imprecise	Likely Likely Likely Likely Likely Likely Likely Likely Likely Likely	Very low Low Low Very low Very low Very low Low Low Very low Very low
		54-month follow up											
Unfavourable outcome	1	Low/Middle education level Comorbidity skeletal system Poor mental Health (SF-36 score <50) >3-month symptom duration Bilateral knee symptoms Self-report warm knee History of non-traumatic knee symptoms Valgus Pain passive flexion Pain passive extension Bony enlargement of joint	[26] [26] [26] [26] [26] [26] [26] [26] [26] [26] [26]	OR OR OR OR OR OR OR OR OR OR OR	2.38 (1.47–3.85) 2.09 (1.34–3.27) 2.81 (1.16–6.83) 2.45 (1.51–3.98) 2.80 (1.73–4.51) 2.36 (1.61–3.68) 3.39 (2.03–5.65) 2.25 (1.38–3.67) 2.44 (1.51–3.94) 2.27 (1.40–3.71) 3.05 (1.38–6.72)	<0.01 <0.01 0.02 <0.01 <0.01 <0.01 <0.01 <0.01 <0.01 <0.01 0.01	1.94 (1.18–3.19) 1.79 (1.12–2.87) **2.95 (1.16–7.48)** 2.20 (1.27–3.78) 1.89 (1.11–3.19) 2.07 (1.27–3.36) **2.59 (1.52–4.41)** 2.07 (1.24–3.48) 1.94 (1.17–3.21) **1.72 (1.01–2.92)** **2.64 (1.17–5.96)**	0.01 0.02 0.02 0.01 0.02 <0.01 <0.01 0.01 <0.01 0.05 0.02	- - - - - - - - - - -	Small Small Moderate Small Small Small Moderate Small Small Small Moderate	Imprecise Imprecise Imprecise Imprecise Imprecise Imprecise Imprecise Imprecise Imprecise Imprecise Imprecise	Likely Likely Likely Likely Likely Likely Likely Likely Likely Likely Likely	Very low Very low Low Very low Very low Very low Low Very low Very low Very low Low
Self-reported perceived recovery		12-month follow up											
	2	Age Poor general health (SF 36 < 50) History of non-traumatic knee symptoms Floating patella	[28] [28] [28] [28]	OR OR OR OR	1.03 (1.01–1.05) 2.64 (0.93–7.52) 1.94 (1.14–3.28) 0.52 (0.30–0.91)	<0.001 <0.07 0.01 0.02	1.03 (1.01–1.05) **3.10 (1.18–8.16)** 1.94 (1.14–3.28) **0.48 (0.27–0.84)**	<0.001 0.02 0.01 0.01	- - - -	Small Moderate Small Moderate	Imprecise Imprecise Imprecise Imprecise	Likely Likely Likely Likely	Very low Very low Very low Very low
		54-month follow up											
		Age BMI >27 non MSK comorbidity Self-reported crepitus History of non-traumatic knee symptoms	[28] [28] [28] [28] [28] [31]	OR OR OR OR OR HR	1.04 (1.01–1.06) 3.30 (1.72–6.32) 2.14 (0.98–4.69) 3.28 (1.65–6.49) 2.96 (1.53–5.73) 0.47 (0.30–0.74)	<0.01 <0.01 0.06 <0.01 <0.00 <0.20	1.03 (1.01–1.05) **2.86 (1.44–5.68)** 2.40 (1.04–5.57) 2.22 (1.38–3.59) 2.28 (1.15–4.53) **0.51 (0.33–0.81)**	0.02 <0.001 0.04 0.00 0.02 <0.001	- - - - Yes Yes	Small Moderate Small Small Small Moderate	Imprecise Imprecise Imprecise Imprecise Imprecise Imprecise	Likely Likely Likely Likely Likely Likely	Very low Very low Very low Very low Low Low
		Three-month follow up											
Poor functional outcome	3	Age Female Duration of knee complaint WOMAC pain WOMAC functioning PCI sub-scale [3]; distress high *vs.* lowest tertitle Complaints of upper and lower extremity *vs.* knee only complaint	[31] [31] [31] [31] [31] [31] [31]	RC RC RC RC RC RC RC	−0.29 (−0.48 to 0.09) - −3.74 (−5.57 to 1.91) 0.33 (0.19–0.48) 0.47 (0.35–0.59) - −6.72 (−13.26 to 0.18)	<0.20 <0.20 <0.20 <0.20 <0.20 <0.20 <0.20	−0.21 (−0.36 to −0.06) **−8.00 (**−**12.53 to −3.46)** **−2.58 (−4.01 to −1.15)** −0.21 (0.39–0.04) 0.82 (0.66–0.99) −17.40 (−29.10 to −5.70) −5.19 (−10.36 to −0.03)	0.01 <0.001 <0.001 0.02 <0.001 <0.001 0.05	- - - - - - -	Small Large Moderate Small Small Large Large	Imprecise Imprecise Imprecise Imprecise Imprecise Imprecise Imprecise	Likely Likely Likely Likely Likely Likely Likely	Very low Very low Low Very low Very low Low Low
		12-month follow up											
		Age Duration of knee complaint WOMAC stiffness WOMAC functioning PCI sub-scale [4]; retreating mid *vs.* low PCI sub-scale [2]; distraction high *vs.* low	[31] [31] [31] [31] [31] [31]	RC RC RC RC RC RC	−0.29 (−0.48 to 0.09) −3.74 (−5.57 to 1.91) 0.14 (0.02–0.25) 0.47 (0.35–0.59) 11.48 (3.48–19.48 −24.35 (−41.25 to 7.46)	<0.20 <0.20 <0.20 <0.20 <0.20 <0.20	−0.29 (−0.45 to 0.12) **−2.71 (−4.19 to −1.24** −0.16 (−0.29 to −0.03 0.65 (0.50–0.80) **6.54 (0.18–12.89)** **−28.16 (−42.41 to −13.90)**	<0.001 <0.001 0.02 <0.001 0.04 <0.001	- - - - - -	Small Moderate Small Small Large Large	Imprecise Imprecise Imprecise Imprecise Imprecise Imprecise	Likely Likely Likely Likely Likely Likely	Very low Low Very low Very low Low Low
		18-month follow up											
		Age 60–69 Age 70+ BMI 25–29.9 BMI > 30 Possible anxiety Probable anxiety Chronic pain grade II Chronic pain grade III Duration of morning stiffness <30 min Local tender point count 2 Local tender point count 3 Local tender point count 4–6 Single leg stand 10–29 s Single leg stand 4–9 s Single leg stand <4 s	[29] [29] [29] [29] [29] [29] [29] [29] [30] [30] [30] [30] [30] [30] [30]	RR RR RR RR RR RR RR RR RR RR RR RR RR RR RR	1.39 (1.08– 1.77) 1.34 (1.02–1.77) 1.58 (1.11–2.26) 1.82 (1.28–2.60) 1.40 (1.11–1.76) 1.52 (1.12–2.07) 1.39 (1.09–1.76) 1.80 (1.30–2.49) 1.68 (1.33–2.13) 1.51 (1.12–2.05) 1.66 (1.21–2.27) 1.63 (1.20–2.23) 1.34 (0.97–1.83) 1.61 (1.20–2.15) 1.65 (1.21–2.24)	0.012 0.038 0.011 0.001 0.005 0.007 0.008 <0.001 <0.001 0.008 0.002 0.002 0.072 0.001 0.001	**1.38 (1.06–1.80)** **1.44 (1.08–1.92)** **1.50 (1.04–2.15)** **1.64 (1.14–2.38)** **1.43 (1.06–1.71)** **1.44 (1.04–1.98)** **1.34 (1.05–1.71)** **1.55 (1.10–2.1** **1.47 (1.13–1.89)** **1.45 (1.06–1.96)** **1.54 (1.12–2.12** **1.48 (1.07–2.04)** **1.27 (0.92–1.74)** **1.50 (1.12– 2.01)** **1.49 (1.09–2.04)**	0.017 0.014 0.029 0.008 0.015 0.027 0.023 0.013 0.004 0.018 0.008 0.017 0.146 0.007 0.014	- - - - - - - - - - - - - - -	Small Small Small Small Small Small Small Small Small Small Small Small Small Small Small	Imprecise Imprecise Imprecise Imprecise Imprecise Imprecise Imprecise Imprecise Imprecise Imprecise Imprecise Imprecise Imprecise Imprecise Imprecise	Likely Likely Likely Likely Likely Likely Likely Likely Likely Likely Likely Likely Likely Likely Likely	Very low Very low Very low Very low Very low Very low Very low Very low Very low Very low Very low Very low Very low Very low Very low

Key: Pain Coping Inventory (PCI (strategy number used); Body Mass Index (BMI); Musculoskeletal (MSK); Passive Range of Movement (PROM); Western Ontario and McMaster Universities Osteoarthritis Index (WOMAC); Regression Coefficient (RC); Risk Ratio (RR); Odds Ratio (OR); Confidence Interval (CI). RC interpretation: Value >0 = greater reduction in pain/improved function; <0 = less reduction in pain or functioning. RC classification of effect size: Small if measures between -1.4 to 0 and 0 to 1.4, moderate -1.41 to -3.4 and 1.4 to 3.4, large > -3.41 and >3.41. OR/HR interpretation: Value >1 = Increased association; 1 = no association; <1 to a limit of 0 = reduced association. OR/HR classification of effect size >1: Small if measures between 1 to -1.49, moderate 2.5 to 4.24, large >4.25 (24). OR/HR classification of effect size <1: Small if measures between 0.99 to 0.66, moderate 0.65 to 0.32 and large if <0.32.

Effect size and confidence intervals in **bold** text indicate prognostic value (>moderate effect size with or without narrow CI's or small effect size with narrow CI's)

## Results

### Study selection

**Figure 1.**
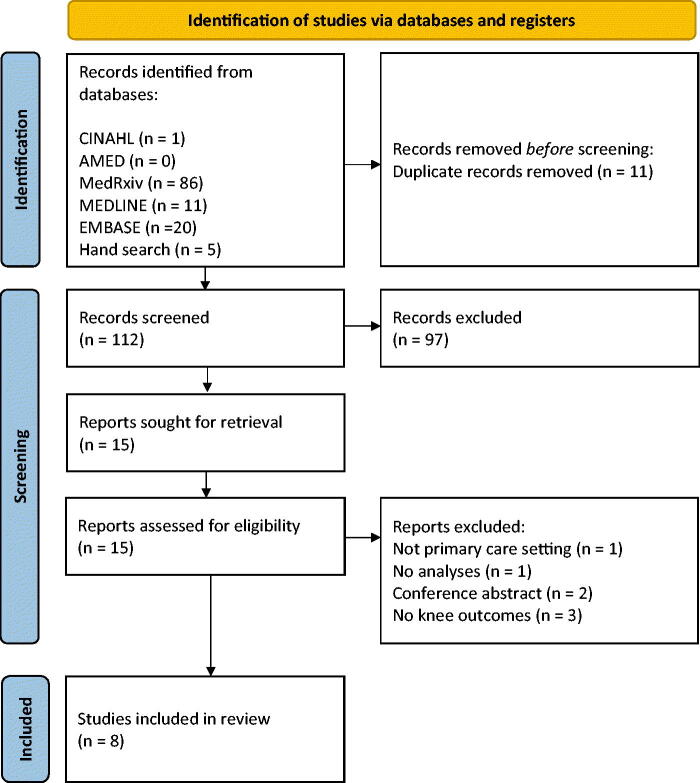
PRISMA flow chart outlining the literature search strategy and study selection process.

The searches returned 123 results with 11 duplicates, leaving 112 studies. After screening titles and abstracts, 97 were excluded. The remaining 15 studies underwent full-text evaluation, where a further seven were excluded. Eight studies were included within the evidence synthesis (Figure 1). All excluded studies are listed, with reasons for exclusion in Supplementary file 9.

### Characteristics and quality of included studies

For all included studies, the characteristics, candidate prognostic factors, outcomes and QUIPS assessments are presented in Table 1. A narrative summary of these elements across studies is provided below.

### General

Included studies were of prospective cohort design. The majority (six) were based in the Netherlands [25–28,31] and the remaining two based in the UK [30,32]. Collection of outcome measures ranged from 54 months [26–28] to 3 months [24–26,29]. Follow-up frequency ranged from five follow up time points in three studies [24,26,28], two in two studies [27,31] and one in the remaining three studies [25,30,32].

### Sample size

None of the included studies specified a sample size calculation or justified the sample size used.

### Participants

The eight included studies had a total of 3872 participants, ranging from 705 (25) to 172 (28). One study did not specify the number of participants according to biological sex [30]. The total male and female participants in the remaining studies were equivalent to 1231 (39.6%) and 1875, respectively (60.4%) [25–29,31].

### Candidate prognostic factors

Demographic factors were investigated in all eight studies, including: age, gender, and BMI [22–29]. Health-related factors were reported in six studies, including: smoking history, skeletal and non-skeletal comorbidities [24,25,27–30]. One study reported on co-morbidities [24]. Knee symptoms and signs were investigated in all eight studies; frequently reported were knee pain level [24–31], Western Ontario and McMaster Universities Osteoarthritis Index (WOMAC) pain, stiffness and function questionnaire [24–27], duration of knee symptoms [25,26,29,31] presence of locking [25,27,28,30] and symptoms of giving way [25,27,28,30]. Physical examination factors were reported in five studies [25–26,28,30] and frequently included palpable warmth [24–26], presence of a joint effusion [26,28,29], and collateral ligament testing [25,26,28]. Physical examination terminology varied. One study gave the name of the tests (both medial and lateral) for collateral ligament testing [25]. Another termed ligament testing as instability [27]. Six studies investigated patient characteristics which commonly related to sport participation [25,28] paid employment [28,31] and marital status [29,31].

Psychosocial factors were investigated in all eight studies, although some specific factors were reported in only one study. Education level was the most frequent reported factor [24–26,28,31]. Three studies investigated coping strategies with pain [24,31] and fear of movement [31] using the six subscales from the pain coping inventory (PCI) and the Tampa Scale for Kinesiophobia respectively. Two studies recorded anxiety [29,30] and two studies recorded sick leave as candidate variables [26,27].

Candidate factors derived from radiological and haematological investigations were infrequent. Two studies used X-ray investigations [24,30], one assessed both the knee and hip [24] and one assessed the knee only [30]. One study included blood markers, specifically erythrocyte sedimentation rate as a potential prognostic factor [22].

### Outcomes

Knee pain outcome measures were reported by two studies [24,31]; 10 and 11 point numerical rating scales (NRS) were used, respectively. Two studies investigated persistent knee symptoms, using several standardized self-reported symptom questionnaires where responses were dichotomized [25,27]. Belo et al. [25] used the WOMAC, the Medical Outcomes Study Short Form 36 Health Survey (SF-36), the Knee Society Score (KSS) function questions, the Lysholm Knee Scoring Scale, the Tampa Scale for Kinesophobia (assessed at baseline) and questions about experience of recovery or worsening. Kastelein et al. [27] used the Knee Society Score, the Lysholm Knee Scoring Scale and the WOMAC.

One study utilized an unfavourable knee outcome, defined as the presence of persistent knee symptoms or having undergone knee replacement surgery during a six year follow up [26]. Two studies assessed self-reported perceived knee recovery [28,31]; one used an ordinal scale that was dichotomized according to whether clinical recovery occurred or not [31]. The other also categorized perceived clinical recovery (completely recovered and much improved versus persistent knee symptoms (slightly improved, no change, slightly worsened, much worsened and worse than ever) [28]. Three studies assessed functional outcomes, using the physical functioning subscale of the WOMAC, lower scores indicated better functioning [29–31]. Six of the included studies reported on outcome validity but not reliability [24–28,31].

### Statistical analysis

The types of statistical analyses used varied across all eight studies. Four studies used logistic regression [24–28], two used cox regression [29,30], one used both cox and linear regression [24,31] and one used latent class growth analysis.

All included studies used univariable screening to inform prognostic factor selection for inclusion in multivariable models, based upon statistical significance values [24–31]. The analyses in five studies further reduced the number of candidate prognostic factors in multivariable models by employing backwards variable selection procedures [25–28,30,31].

### Effect measures

Significant heterogeneity was evident for reported effect estimates, limiting direct comparisons across prognostic factors. Four studies reported ORs [25–28] one reported regression coefficients (RC) and HR [31] and three reported risk ratios (RR) [24,29,30].

### Risk of bias within and across studies

#### General

The overall quality of reporting across studies was variable. Out of 48 domains that were reported across all studies, 11 domains (23%) were classed as having low RoB. Most domains across studies were classed as moderate (20 domains or 42%) or high RoB (17 domains or 35%) (Table 1).

#### Participation

Five studies were classed as low [24–27,29–31] and three of moderate RoB [26–28] in terms of participation reporting. Three did not provide dates of the study recruitment period [26–28]. Those considered low RoB reported on recruitment periods, geographical location and characteristics of the study population. Studies considered of moderate RoB demonstrated variable reporting quality. Key characteristics of the population source and recruitment periods were unclear but other key information such as recruitment place and eligibility criteria were specifically stated for all included studies.

#### Study attrition

Four studies were considered as high RoB as key characteristics of loss and rate of loss to follow-up were either not described or ambiguously reported [26,29–31]. One was considered of moderate risk due to ambiguous reporting of attrition and key characteristics [27]. Three were considered of low risk; there was low attrition rates and specific details provided for loss to follow up, key characteristics of those lost [24,25,28].

#### Prognostic factors

Five studies were considered of moderate RoB [25,26,29–31]. Reliability and validity of prognostic factor measurement methods were not reported; the method of imputation for missing prognostic factor data was also not reported in three studies [29–31].

For candidate factors that consisted of continuous data, the type of variable categorization was not specifically stated in four studies [26,29–31].

The remaining studies were considered high RoB; they did not report missing data, state definitions for categorical data or the reliability of prognostic factor measurement [24,27,28].

#### Outcome measurement

No studies were considered of high RoB with respect to outcome. Four were considered of moderate risk because they did not report validity and/or reliability for outcome measures, a potential source of misclassification bias according to QUIPS criteria [24,25,27,28,31]. The remaining studies were considered low risk with follow up time frames were clearly defined [24,26,29,30]. Two of which described the validity of the outcome measure but did not describe its reliability [29,30].

#### Adjustment for other prognostic factors

We pre-specified that as a minimum, studies should adjust for age and biological sex in their multivariable analyses as these were common to all participants in all studies. Four studies adjusted for the prognostic effect of both, and were considered as moderate RoB [24,26,27,31]. The remaining four studies were considered high RoB because only age was adjusted for but not biological sex [24,26,29,30] and definitions for other prognostic factors used for adjustment were either unclear or not reported [24,26,29,30]. Additionally, handling of missing data was not reported in three of these four studies [24,29,30].

#### Statistical analysis

All included studies used univariable screening to select prognostic factors for inclusion in multivariable models based upon statistical significance. This data-driven approach to prognostic factor selection is generally not recommended for constructing multivariable models, as it may result in some clinically important factors being excluded from final analyses; this means that prognostic effects may not be properly adjusted for. Instead, recent recommendations are that multivariable models should be constructed using prognostic factors identified from the literature and clinical reasoning [10,33]. Therefore, none of the included studies could be considered as low RoB. Five studies were considered high risk, because there was evidence of selective reporting [24,26,29–31]. Only one had a study protocol to make a direct comparison between proposed outcomes and those reported in the full-text publication [26]. Therefore, outcomes listed in the methods section of the remaining studies were compared with those reported in the results section. Although outcomes reported in results were consistent with outcomes specified in methods in all five studies, there was inadequate reporting of non-significant prognostic indicators in the results.

#### Data synthesis

Unfortunately, due to the observed heterogeneity in terms of study methodology, prognostic factors, prognostic effect measures and the large proportion of domains classed as moderate to high RoB, a meta-analysis could not be performed. Instead, a narrative synthesis is presented below. A summary of all significant and insignificant prognostic factors derived from all studies (with their associated effect measures, CIs and *p* values) are listed in Table 2 and Supplementary file 10, respectively. Prognostic factors derived from single studies, or factors that were investigated by more than one study are grouped according to the specific outcome measures investigated.

#### Results of studies

Across all studies and follow up time points, a total of 74 prognostic factors were identified (Table 2). A total of 38 and 63 statistically significant univariable and multivariable prognostic factors were identified, respectively. All evidence was considered to be of low to very low quality according to GRADE criteria [23]. This was due to phase 1 explanatory cohort designs, and almost all prognostic factors were established from single studies. This limited between study comparisons in terms of effect sizes, precision, consistency of results and publication bias.

#### Knee pain

Thirteen statistically significant prognostic factors were identified from one low to very low-quality graded study (Table 2). Eight prognostic factors were related to short-term follow up (3 months), and five related to medium term (12 months) follow up [31]. Eleven were associated with small to moderate effect sizes with narrow and wide CIs respectively which may have prognostic value (Table 2). Statistically significant univariable prognostic associations are unknown because this was not reported, only that univariable factors met a predefined level of significance (*p* < 0.20) to be considered for multivariable analysis.

#### Persistent knee symptoms

**Figure 2.**
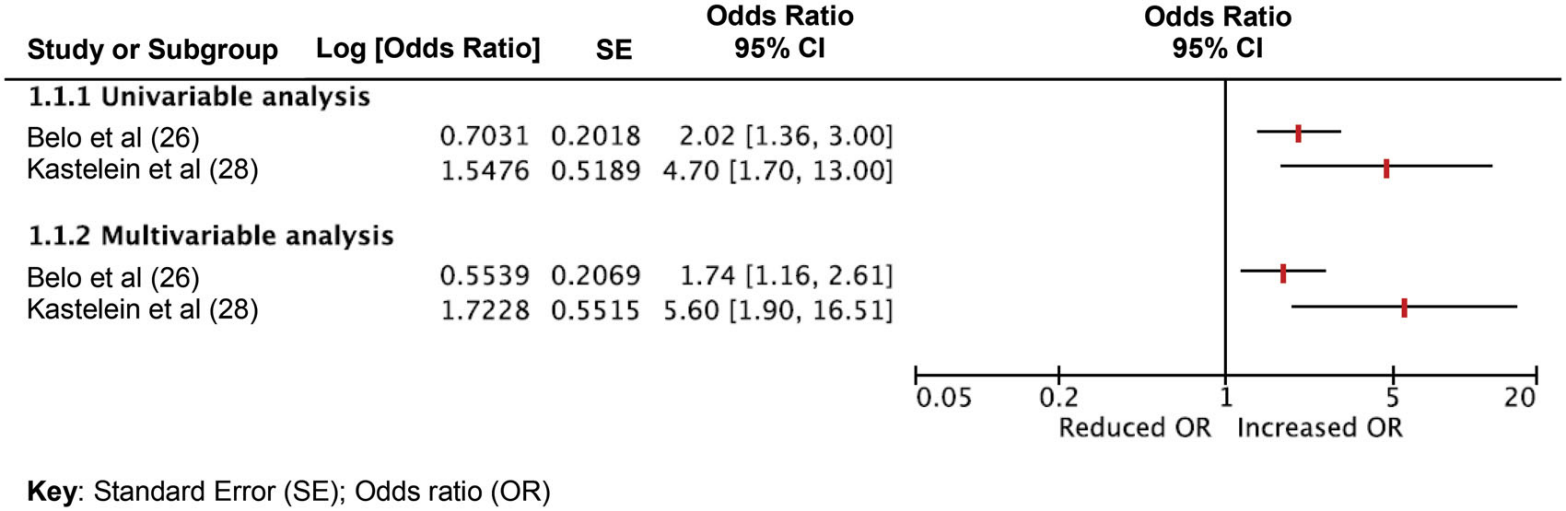
Graph comparing poorer education level as a prognostic factor for persisting knee symptoms in two at 12-month follow up – odds ratio analyses.

**Figure 3.**
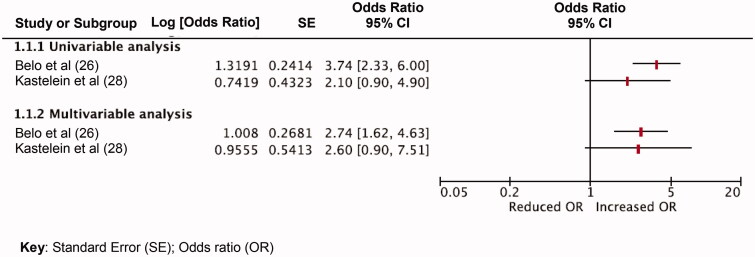
Graph comparing bilateral knee symptoms as a prognostic factor for persisting knee symptoms in two at 12-month follow up – odds ratio analyses.

Ten prognostic factors were identified across two studies [24,27]. There was consensus (in both univariable and multivariable analyses) that poor education level (univariable OR range = 2.02 − 4.70; 95%CI = 1.36–13; *p* value range = 0.002 to <0.20; multivariable OR range = 1.74–5.6; 95%CI range = 1.16–16.2, *p* value = < 0.001) and bilateral knee symptoms (univariable OR range = 2.10–3.74; 95%CI range = 0.90–6.0; multivariable OR range = 2.60–2.74; 95%CI range = 0.90–7.51) were associated with persisting knee symptoms at 12 months (Figures 2 and 3). *p* Values were only reported for one of the two studies (Table 2). Although statistical significance was not reported by Belo et al. [25] in multivariable analysis, age (OR 2.02 95%CI; 1.30–3.13) kinesiophobia (OR 1.85 95%CI; 1.26–2.72) and comorbidity (OR1.50 95%CI; 0.99–2.28) of the MSK system may have provisional prognostic importance.

#### Unfavourable outcome

Eleven statistically significant prognostic factors were identified (in univariable and multivariable analyses) at 54-month follow up [26]. In particular, history of non-traumatic knee symptoms (univariable analysis OR; 3.39 95%CI; 2.03–5.65 *p* < 0.01, multivariable analysis OR; 2.59; 95%CI; 1.52–4.41 *p* = < 0.001); bony enlargement of the knee joint (univariable analysis OR; 3.05 95%CI; 1.38–6.72 *p* = 0.01, multivariable analysis OR; 2.64 95%CI; 1.17–5.96 *p* = 0.02) and poor quality of life (SF-36 score <50) (univariable analysis OR; 2.81 95%CI; 1.16–6.83, *p* = 0.02; multivariable analysis OR; 2.95; 95%CI; 1.16–7.48; *p* = 0.02) demonstrated the greatest associations with unfavourable outcome.

#### Self-reported perceived recovery

Nine prognostic factors were identified. Eight derived from one study [28], seven being statistically associated across both univariable and multivariable analyses. In the short term (≤12 months) poor general health (univariable analysis OR 2.64 95%CI = 0.93–7.52; multivariable analysis OR 3.10 95%CI = 1.18–8.16; *p* = 0.02) and a floating (unsecure) patella (univariable analysis OR 0.52 95%CI = 0.30–0.91; multivariable analysis OR 0.48 95%CI = 0.48–0.84 *p* = 0.02) demonstrated moderate effect sizes with corresponding large and narrow CIs, respectively, associated with poorer self-reported perceived recovery [28]. In the long term, (six years) body mass index (BMI) >27 (univariable analysis OR 3.30 95%CI = 1.72–6.32 *p* < 0.01; multivariable analysis OR 2.86 95%CI = 1.44–5.68 *p* < 0.001) also demonstrated a moderate effect size [28].

A history of non-traumatic knee symptoms was also identified as a prognostic factor in two studies [9,14]; one utilized ORs (univariable analysis OR; 2.96 95%CI = 1.53–5.73 *p* = < 0.001 (multivariable analysis OR; 2.28 95%CI = 1.15–4.53 *p* = 0.02) while the other utilized HRs (univariable analysis HR; 0.47 95%CI = 0.30–0.74 (multivariable analysis HR; 0.51 95%CI = 0.33–0.81 *p* = < 0.001) therefore preventing direct comparisons. Additionally, although they did not reach statistical significance for single studies within which they were tested, laxity on anterior drawer testing (univariable analysis OR; 1.70 95%CI = 0.84–3.30 *p* = 0.05 (multivariable analysis OR; 1.68 95%CI = 0.98 − 2.88 *p* = 0.06) and a popliteal fossa effusion (univariable analysis RR; 1.61 95%CI = 0.91–2.84 *p*= 0.10 (multivariable analysis RR 1.68 95%CI = 0.94–3.03 *p* = 0.08) may have some prognostic (Supplementary file 10) importance [27].

#### Poor functional outcome

Over varying follow up times, 28 statistically significant multivariable prognostic factors were identified from three single studies [29–31]. At 3 months, 7 were identified [31]. Longer duration of knee complaint (univariable analysis regression coefficient (RC) −3.74 95%CI = −5.57 to 1.91 *p* < 0.20; multivariable analysis RC; −2.58 95%CI = −4.01 to −1.15 *p* < 0.001) and female biological sex (multivariable analysis RC; −8.00 95%CI = −12.53 to −3.46 *p* < 0.001), were associated with poorer functional outcome with moderate and large effect sizes, respectively.

At 12 months, six factors were identified [31]. Longer duration of knee complaint (univariable analysis RC −3.74 95%CI = −5.57 to 1.91; multivariable analysis RC; −2.71 95%CI = −4.19 to −1.24 *p* < 0.001); middle and higher pain catastrophising scores on the PCI (retreating sub-scale) questionnaire (univariable analysis RC 11.48 95%CI = 3.48–19.48; multivariable analysis RC 6.54 95%CI = 0.18–12.89 *p* = 0.04) and lower pain coping on the PCI (distraction sub-scale) questionnaire (univariable analysis RC 24.35 95%CI= −41.25 to 7.46; multivariable analysis RC; −28.16 95%CI= −42.41 to −13.90 *p* < 0.001) were associated with worse functional outcomes with moderate, large and large multivariable effect sizes, respectively (Table 2).

At 18 months, there were 15 statistically significant prognostic factors consistent in both univariable and multivariable analysis derived from two studies that were associated with outcome with narrow CIs [29,30]. Finally, while the presence of bilateral knee pain (RR 1.28 95%CI = 0.98–1.68 *p* = 0.068) and morning stiffness lasting >30 min (RR 1.55 95%CI = 0.99–2.43 *p* = 0.057) were classed as non-significant in multivariable analysis (Supplementary file 10), they may still have some limited prognostic importance due to moderate effect sizes [28].

## Discussion

This review has summarized, appraised and synthesized the evidence to identify prognostic factors associated with changes in outcomes relevant to knee pain in adult patients, using data obtained from initial primary care consultations.

All evidence included in this review was low or very low quality according to the modified GRADE assessment (Table 2). This could be explained in part because all included studies were described as phase 1 prognostic studies, i.e. studies that have exclusively sought to identify and explore any potential associations between outcomes and candidate prognostic factors [34]. Consequently, when using the modified GRADE criteria, a moderate quality of evidence was the maximum score that could be obtained. Studies were then downgraded if there was evidence of imprecision (including absence of sample size calculation) and inconsistency of results, where associations have not been confirmed in other studies [23]. In particular, between-study heterogeneity limited the number of comparisons that could be made in terms of effect measures, follow up time points, candidate prognostic factors and outcome measures. It is clear that further research is required to provide evidence of the consistency of these results across other cohorts.

A significant issue identified was related to the general conduct of multivariable analyses. To establish the independent association of a prognostic factor and an outcome, analyses should be adjusted for other important prognostic factors that may otherwise distort the true relationship [14,16]. It has been suggested that age [19,35,36] and biological sex [37–39] have previously been shown to be associated with worsening knee outcomes. Consequently, we stated a priori (PROSPERO) registration ID; CRD42021229699) that these should be essential factors used for adjustment purposes, as these are common to all participants and thus may have an influence on prognostic estimates through mechanisms such as confounding, mediation and moderation [11]. However, only four studies adjusted for both the prognostic effects of age and biological sex [25–27,31]. Instead, univariable screening was commonly used to select candidate prognostic factors for inclusion in multivariable models, based upon statistical significance [10,17]. While this may have been acceptable practice previously, it is unlikely that models were adjusted appropriately using other clinically important prognostic factors. Indeed, current recommendations suggest that candidate factors should be selected for inclusion based upon existing evidence and clinical reasoning, to ensure all important factors are considered [17]. Several included papers [24,26,29–31] were appraised as low or very low quality using QUIPS.

Whilst we acknowledge that these papers might have been considered as high quality at the time of publication, the introduction and advancement of reporting guidelines (such as the Reporting recommendations for tumour MARKer prognostic studies [40] and appraisal guidelines (such as QUIPS) in response to evolving best practice means that unfortunately, these papers inevitably fall short of current required standards. Importantly though, these papers have provided an essential foundation to underpin advancements in primary care prognostic research.

Despite the low quality of graded evidence, there was consensus from two studies that a lower education level and bilateral knee symptoms were independently associated with an increased odds of persistent knee pain at 12-month follow up [25,27]. This has potential clinical importance for healthcare practitioners working in a primary care setting because patients who present with bilateral knee pain that have a lower educational background at initial consultation may have greater odds of longer-term symptoms. However, it must be remembered that because of the low overall quality of the evidence, the prognostic value of these factors should only be considered provisional to be confirmed in future studies. Despite their relatively limited clinical value, in terms of further prognosis research, these prognostic factors would be suitable for inclusion in any future studies to develop a prognostic model to predict changes in knee pain over time.

Our results are consistent with other similar reviews of generic prognostic factors MSK outcomes in primary care [20] and prognostic factors for the shoulder joint in secondary care [41] which have both suggested caution in their conclusions due to selective reporting, poor control of confounding, bias in study design and small sample sizes within primary studies. We found that some of the potential prognostic factors identified from low-quality studies were consistent with those observed for changes in knee pain outcomes in secondary care [18,19]. Specifically, these factors (Table 2) include increased age [26,29], increased body mass [22,26,27,29] and previous knee injury [24,29]. We also found that some prognostic factors identified from single, low-quality studies were also consistent with prognostic factors for generic MSK pain outcomes observed in primary care [32]. These factors (Table 2) that may have importance include higher pain severity at baseline [31], longer pain duration [31], multiple-site pain [24,26], anxiety and/or depression [29,31], adverse coping strategies [31] and older age [26,29]. Nevertheless, because of the similar issues afflicting the quality of the wider evidence base, any consistency between our findings and these studies should be interpreted with caution. There is a need for a greater number of well-conducted studies to further our understanding related to prognostic factors and their relationship with knee pain in both primary and secondary care settings.

Finally, we found that six of the eight included studies were based in the Netherlands [25–28,31]. The south of the Netherlands is particularly prone to significant land rise [42] and previously, a mountainous landscape was found to be an independent prognostic factor for knee pain [43]. How generalizable the current review findings are to other nationalities with flatter gradients is uncertain. Further high-quality exploratory and confirmatory prognostic factor studies are required that utilize large cohorts of primary care patients based in other countries.

## Limitations

Our review only considered peer-reviewed published studies and pre-prints from the MedRxiv database. An extensive search of conference abstracts and other grey literature was not conducted, which may have inadvertently introduced some publication bias [44,45].

The QUIPS appraisal tool was used as it is specific to prognostic research for systematic reviews and has been demonstrated to have high reliability [21]. However, we did not formally establish inter-rater reliability between reviewers.

## Conclusion

This is the first systematic review that has investigated candidate prognostic factors identified from data collected at initial primary care consultation, and associations with changes in outcomes for patients with knee pain. Results from two papers suggest that the presence of bilateral knee pain and a lower educational level were independently associated with persisting knee pain at 12-month follow up. However, this must be interpreted with caution because results obtained are derived from a pool of low to very low quality of evidence. Other factors were identified as having potential associations with various knee pain outcome measures, but all were derived from single studies. Further research is essential to improve the knowledge base of this important area of primary care MSK research [46].

## Supplementary Material

Supplemental Material

Supplemental Material

Supplemental Material

Supplemental Material

Supplemental Material

Supplemental Material

Supplemental Material

Supplemental Material

Supplemental Material

## Data Availability

Data sharing is not applicable to this article as no new data were created or analysed in this study.

## References

[1] Jin Z, Wang D, Zhang H, et al. Incidence trend of five common musculoskeletal disorders from 1990 to 2017 at the global, regional and national level: results from the global burden of disease study 2017. Ann Rheum Dis. 2020;79(8):1014–1022. 32414807 10.1136/annrheumdis-2020-217050

[2] Kontis V, Bennett JE, Mathers CD, et al. Future life expectancy in 35 industrialised countries: projections with a bayesian model ensemble. Lancet. 2017;389(10076):1323–1335. 28236464 10.1016/S0140-6736(16)32381-9 PMC5387671

[3] Institute for Health Metrics and Evaluation. Findings from the Global Burden of Disease Study 2017 | Institute for Health Metrics and Evaluation (IHME). Findings from the Global Burden of Disease Study 2017.Seattle, WA: IHME, 2018.

[4] Dey P, Callaghan M, Cook N, et al. A questionnaire to identify patellofemoral pain in the community: an exploration of measurement properties. BMC Musculoskelet Disord. 2016;17(1):237. 27245443 10.1186/s12891-016-1097-5 PMC4886395

[5] Urwin M, Symmons D, Allison T, et al. Estimating the burden of musculoskeletal disorders in the community: the comparative prevalence of symptoms at different anatomical sites, and the relation to social deprivation. Ann Rheum Dis. 1998;57:649–655. 9924205 10.1136/ard.57.11.649 PMC1752494

[6] Jordan KP, Kadam UT, Hayward R, et al. Annual consultation prevalence of regional musculoskeletal problems in primary care: an observational study. BMC Musculoskelet Disord. 2010;11:144. 20598124 10.1186/1471-2474-11-144 PMC2903510

[7] Hughes C, Bleakley C. Treatment of knee pain in primary care: pharmacists and physiotherapists need to be a part of the team. BMJ. 2006;333(7576):981–982. 17095761 10.1136/bmj.39024.417813.BE PMC1635645

[8] Frese T, Peyton L, Mahlmeister J, et al. Knee pain as the reason for encounter in general practice. ISRN Family Med. 2013;2013:1–6. 10.5402/2013/930825 PMC4041260 24959577

[9] Porcheret M, Jordan K, Jinks C. Treatment of knee pain in older adults in primary care: development of an evidence-based model of care. Rheumatology. 2007;46(11):1694–1700. 17938135 10.1093/rheumatology/kem232

[10] Bullock GS, Hughes T, Sergeant JC, et al. Methods matter: clinical prediction models will benefit sports medicine practice, but only if they are properly developed and validated. Br J Sports Med. 2021;55(23):1319–1321. 34215643 10.1136/bjsports-2021-104329

[11] Riley RD, Moons KGM, Snell KIE, et al. A guide to systematic review and meta-analysis of prognostic factor studies. BMJ. 2019;364:k4597. 30700442 10.1136/bmj.k4597

[12] Hingorani AD, Windt DAVD, Riley RD, et al. Prognosis research strategy (PROGRESS) 4: stratified medicine research. BMJ. 2013;346(feb05 1):e5793–e5793. 23386361 10.1136/bmj.e5793 PMC3565686

[13] Hemingway H, Croft P, Perel P, et al. Prognosis research strategy (PROGRESS) 1: a framework for researching clinical outcomes. BMJ. 2013;346(feb05 1):e5595–e5595. 23386360 10.1136/bmj.e5595 PMC3565687

[14] Riley RD, Hayden JA, Steyerberg EW, et al. Prognosis research strategy (PROGRESS) 2: prognostic factor research. PLoS Med. 2013;10(2):e1001380. 23393429 10.1371/journal.pmed.1001380 PMC3564757

[15] Riley RD, van der Windt D, Peter Croft KGMM. Prognosis research in healthcare: concepts, methods, and impact. Oxford: Oxford University Press; 2019.

[16] Steyerberg EW, Moons KGM, van der Windt DA, et al. Prognosis research strategy (PROGRESS) 3: prognostic model research. PLoS Med. 2013;10(2):e1001381. 23393430 10.1371/journal.pmed.1001381 PMC3564751

[17] Moons KGM, Altman DG, Reitsma JB, et al. Transparent reporting of a multivariable prediction model for individual prognosis or diagnosis (TRIPOD): explanation and elaboration. Ann Intern Med. 2015;162(1):W1–W73. 25560730 10.7326/M14-0698

[18] Silverwood V, Blagojevic-Bucknall M, Jinks C, et al. Current evidence on risk factors for knee osteoarthritis in older adults: a systematic review and meta-analysis. Osteoarthritis Cartilage. 2015;23(4):507–515. 25447976 10.1016/j.joca.2014.11.019

[19] Miranda H, Viikari-Juntura E, Martikainen R, et al. A prospective study on knee pain and its risk factors. Osteoarthritis Cartilage. 2002;10:623–630. 12479384 10.1053/joca.2002.0796

[20] Artus M, Campbell P, Mallen CD, et al. Generic prognostic factors for musculoskeletal pain in primary care: a systematic review. BMJ Open. 2017;7(1):e012901. 10.1136/bmjopen-2016-012901 PMC5253570 28096253

[21] Hayden JA, van der Windt DA, Cartwright JL, et al. Assessing bias in studies of prognostic factors. Ann Intern Med. 2013;158(4):280. 23420236 10.7326/0003-4819-158-4-201302190-00009

[22] Grooten WJA, Tseli E, Äng BO, et al. Elaborating on the assessment of the risk of bias in prognostic studies in pain rehabilitation using QUIPS—aspects of interrater agreement. Diagn Progn Res. 2019;3(1):5. 31093575 10.1186/s41512-019-0050-0 PMC6460536

[23] Huguet A, Hayden JA, Stinson J, et al. Judging the quality of evidence in reviews of prognostic factor research: adapting the GRADE framework. Syst Rev. 2013;2(1):71. 24007720 10.1186/2046-4053-2-71 PMC3930077

[24] Bastick AN, Wesseling J, Damen J, et al. Defining knee pain trajectories in early symptomatic knee osteoarthritis in primary care: 5-year results from a nationwide prospective cohort study (CHECK). Br J Gen Pract. 2016;66(642):e32–e39. 26639946 10.3399/bjgp15X688129 PMC4684033

[25] Belo JN, Berger MY, Koes BW, et al. Prognostic factors in adults with knee pain in general practice. Arthritis Rheum. 2009;61:143–151. 19177535 10.1002/art.24419

[26] Kastelein M, Luijsterburg PAJ, Belo JN, et al. Six-year course and prognosis of nontraumatic knee symptoms in adults in general practice: a prospective cohort study. Arthritis Care Res (Hoboken). 2011;63(9):1287–1294. 21671415 10.1002/acr.20522

[27] Kastelein M, Luijsterburg PAJ, Heintjes EM, et al. The 6-year trajectory of non-traumatic knee symptoms (including patellofemoral pain) in adolescents and young adults in general practice: a study of clinical predictors. Br J Sports Med. 2015;49(6):400–405. 25431450 10.1136/bjsports-2014-093557

[28] Kastelein M, Luijsterburg PAJ, Verhaar JAN, et al. Six-year course and prognosis of traumatic knee symptoms in general practice: cohort study. Eur J Gen Pract. 2016;22(1):23–30. 26653667 10.3109/13814788.2015.1109075

[29] Mallen CD, Peat G, Thomas E, et al. Predicting poor functional outcome in community-dwelling older adults with knee pain: prognostic value of generic indicators. Ann Rheum Dis. 2007;66:1456–1461. 17456527 10.1136/ard.2006.067975 PMC2111642

[30] Thomas E, Peat G, Mallen C, et al. Predicting the course of functional limitation among older adults with knee pain: do local signs, symptoms and radiographs add anything to general indicators? Ann Rheum Dis. 2008;67:1390–1398. 18245111 10.1136/ard.2007.080945

[31] van der Waal JM, Bot SDM, Terwee CB, et al. Course and prognosis of knee complaints in general practice. Arthritis Rheum. 2005;53:920–930. 16342106 10.1002/art.21581

[32] Mallen CD, Peat G, Thomas E, et al. Prognostic factors for musculoskeletal pain in primary care: a systematic review. Br J Gen Pract. 2007;57:655–661. 17688762 PMC2099673

[33] Bullock GS, Collins GS. Improving clinical prognostic model methodology: letter to the editor. Am J Sports Med. 2021;49:NP23–NP25. 33929882 10.1177/03635465211005721

[34] Hayden JA, Côté P, Steenstra IA, et al. Identifying phases of investigation helps planning, appraising, and applying the results of explanatory prognosis studies. J Clin Epidemiol. 2008;61:552–560. 18471659 10.1016/j.jclinepi.2007.08.005

[35] Ding C, Cicuttini F, Scott F, et al. Association between age and knee structural change: a cross sectional MRI based study. Ann Rheum Dis. 2005;64(4):549–555. 15769915 10.1136/ard.2004.023069 PMC1755432

[36] Nguyen USDT, Zhang Y, Zhu Y, et al. Increasing prevalence of knee pain and symptomatic knee osteoarthritis: survey and cohort data. Ann Intern Med. 2011;155(11):725. 22147711 10.1059/0003-4819-155-11-201112060-00004 PMC3408027

[37] Hanna FS, Teichtahl AJ, Wluka AE, et al. Women have increased rates of cartilage loss and progression of cartilage defects at the knee than men: a gender study of adults without clinical knee osteoarthritis. Menopause. 2009;16(4):666–670. 19598333 10.1097/gme.0b013e318198e30e

[38] Cho HJ, Chang CB, Yoo JH, et al. Gender differences in the correlation between symptom and radiographic severity in patients with knee osteoarthritis. Clin Orthop Relat Res. 2010;468:1749–1758. 20204559 10.1007/s11999-010-1282-z PMC2881984

[39] Manninen P, Riihimäki H, Heliövaara M, et al. Overweight, gender and knee osteoarthritls. Int J Obes. 1996;20(6):595–597. 8782738

[40] Sauerbrei W, Taube SE, McShane LM, et al. Reporting recommendations for tumor marker prognostic studies (REMARK): an abridged explanation and elaboration. PLoS Med. 2018;9:e1001216. 10.1371/journal.pmed.1001216 PMC3362085 22675273

[41] Chester R, Shepstone L, Daniell H, et al. Predicting response to physiotherapy treatment for musculoskeletal shoulder pain: a systematic review. BMC Musculoskelet Disord. 2013;14:203. 23834747 10.1186/1471-2474-14-203 PMC3717132

[42] Hollander IG. In the Dutch mountains. L2 J. 2014;6(1):9–13.

[43] Ann K, Kshetri D, Selfe J, et al. Prevalence of knee pain di + ers across ecological landscapes of the Western development region of Nepal. J Quant Res Rehabil Med. 2019;1(3):73–77.

[44] Collier T, Roadley-Battin M, Darlow C, et al. Analysis of conference abstract-to-publication rate in UK orthopaedic research. BMJ Evid Based Med. 2018;23:7–11. 10.1136/ebmed-2017-110831 29367317

[45] Dunn KM, Jordan K, Lacey RJ, et al. Patterns of consent in epidemiologic research: evidence from over 25,000 responders. Am J Epidemiol. 2004;159:1087–1094. 15155293 10.1093/aje/kwh141

[46] Segal NA, Curtis JR, Niu J, et al. Greater trochanteric pain syndrome: epidemiology and associated factors. Arch Phys Med Rehabil. 2007;88(8):988–992. 17678660 10.1016/j.apmr.2007.04.014 PMC2907104

