# Supplementary material for: Prognostic factors associated with changes in knee pain outcomes, identified from initial primary care consultation data. A systematic literature review
Source: Ann Med. 2023 Jan 27;55(1):401–18. doi: 10.1080/07853890.2023.2165706 (PMC9888457; doi:10.1080/07853890.2023.2165706)
Supplement: Supplemental Material [file IANN_A_2165706_SM7703.docx]

**Supplementary file 10: Summary of synthesis of insignificant prognostic factors with associated GRADE evaluation.**

| **Summary of synthesis of prognostic factors** | | | | | | | | |  | **Adapted GRADE Criteria** | | | | | |
| --- | --- | --- | --- | --- | --- | --- | --- | --- | --- | --- | --- | --- | --- | --- | --- |
| Specific outcome | Number of Studies | Potential Prognostic Factors | Authors | Effect Measure | Univariable effect size  (95% CI) | P-value | Multivariable effect size  (95% CI) | P-value |  | Study Limitations | Consistency of results | Effect size | Precision of results | Publication bias | Overall Quality |
| Three-month follow up | | | | | | | | |  |  | | | | | |
| Pain (NRS) | 1 | Cause, overload during usual activities  Distress-middle vs lowest tertitle | Van der Waal et al (30)  Van der Waal et al (30) | RC  RC | 0.56 (-0.28 to 1.40)  0.38 (-0.50 to 1.27) | <0.20  <0.20 | **0.67 (-0.04 to 1.37**  **0.44 (-0.29 to 1.16)** | 0.06  0.24 |  | Many | -  - | Small  Moderate | Imprecise  Imprecise | Likely  Likely | Very Low  Very Low |
| Poor functional outcome | 1 | PCI sub scale (3) reducing demands mid Vs. low  PCI sub scale (3) reducing demands high Vs. low  PCI sub scale 5) distress mid Vs. low  Coexisting complaint lower extremity Vs knee only complaint  Meeting ACSM position stand Vs not meeting norm  Social support | Van der Waal et al (30)  Van der Waal et al (30)  Van der Waal et al (30)  Van der Waal et al (30)  Van der Waal et al (30)  Van der Waal et al (30) | RC  RC  RC  RC  RC  RC | 1.37 (-6.74 to 9.48)  7.31 (0.46 to 14.16)  3.12 (-4.35 to 10.59)  -8,21 (-19.69 to 3.28)  6.66 (-1.25 to 14.58)  -0.64 (-1.05 to 0.22) | <0.20  <0.20  <0.20  <0.20  <0.20  <0.20 | **-5.79 (-11.01 to 0.34)**  -0.39 (-5.54 to 4.76)  0.29 (-5.38 to 5.95)  **-2.65 (-11.52 to 6.22)**  **5.83 (0.20 to 11.87)**  **-0.30 (0.63 to 0.03)** | 0.06  0.88  0.92  0.56  0.06  0.08 |  | Many | -  -  -  -  -  - | Large  Small  Small  Moderate  Large  Small | Imprecise  Imprecise  Imprecise  Imprecise  Imprecise  Imprecise | Likely  Likely  Likely  Likely  Likely  Likely | Low  Very Low  Very Low  Low  Low  Low |
| 12-month follow up | | | | | | | | |  |  | | | | | |
| Pain (NRS) | 1 | Cause, injury during exercise  PCI distraction mid Vs. low  PCI distress mid Vs. low | Van der Waal et al (30)  Van der Waal et al (30)  Van der Waal et al (30) | RC  RC  RC | -1.19 (-0.20 to 2.58)  -1.01 (-0.98 to 0.96)  -0.20 (-1.18 to 0.78) | -  -  - | **0.98 (0.12 to 2.08)**  **-0.32 (-1.10 to 0.47)**  **-0.34 (-1.15 to 0.48)** | 0.08  0.43  0.42 |  | Many | -  -  - | Small  Large  Large | Imprecise  Imprecise  Imprecise | Likely  Likely  Likely | Very Low  Very Low  Very Low |
| Persisting knee symptoms | 2 | BMI >25  Self-reported crepitus knee  History of traumatic knee symptoms | Kastelein et al (26)  Kastelein et al (26)  Belo at al (29) | OR  OR  OR | 2.5 (1.0 to 6.2)  0.4 (0.1 to 1.3)  2.26 (1.47 to 3.46) | 0.04  0.12  <0.20 | **3.2 (1.2 to 8.6)**  **0.4 (0.1 to 1.2)**  **1.56 (0.97-2.49)** | 0.23  0.10  - |  | Many | -  -  - | Moderate  Moderate  Small | Imprecise  Imprecise  Imprecise | Likely  Likely  Likely | Very low  Very low  Very low |
| Unfavourable outcome | 1 | Limitation during daily function (WOMAC >20)  Comorbidity skeletal system | Kastelein et al (25)  Kastelein et al (25) | OR  OR | 1.64 (1.05 to 2.55)  2.09 (1.34 to 3.27) | 0.03  <0.01 | **1.52 (0.93 to 2.49)**  **1.67 (0.98 to 2.84)** | 0.09  0.06 |  | Many | -  - | Small  Small | Imprecise  Imprecise | Likely  Likely | Very low  Very low |
| Self-reported perceived recovery | 1 | Anterior drawer test laxity  Effusion popliteal fossa | Kastelein et al (27)  Kastelein et al (27) | OR  PR | 1.70 (0.84 to 3.30)  1.61 (0.91 to 2.84) | 0.05  0.10 | **1.68 (0.98 to 2.88**  **1.68 (0.94 to 3.03)** | 0. 06  0.08 |  | Many | -  - | Small  Small | Imprecise  Imprecise | Likely  Likely | Very low  Very low |
| Poor functional outcome | 3 | PCI sub-scale (4); retreating high Vs. low  PCI sub-scale (2); distraction mid Vs. low  Chronic pain grade IV  Bilateral Knee pain present  Inactivity gelling present  Duration of morning stiffness >30 min  Local tender point count 1 | Van der Waal et al (30)  Van der Waal et al (30)  Mallen et al (28)  Thomas et al (29)  Thomas et al (29)  Thomas et al (29)  Thomas et al (29) | RC  RC  RR  RR  RR  RR  RR | 6.92 (-0.11 to 13.95)  2.53 (-5.22 to 10.28)  1.75 (1.27 to 2.41)  1.46 (1.12 to 1.90)  1.68 (1.26 to 2.25)  1.84 (1.19 to 2.84)  1.20 (0.88 to 1.65) | 0.001  0.005  <0.001  0.006  0.252 | **2.61 (-3.11 to 8.33)**  **-1.72 (-7.88 to 4.45)**  **1.42 (1.00 to 2.03)**  **1.28 (0.98 to 1.68)**  **1.34 (0.98 to 1.83)**  **1.55 (0.99 to 2.43)**  1.16 (0.84 to 1.58) | 0.37  0.59  0.051  0.068  0.067  0.057  0.370 |  | Many | -  -  -  -  -  -  - | Moderate  Large  Small  Small  Small  Small  Small | Imprecise  Imprecise  Imprecise  Imprecise  Imprecise  Imprecise  Imprecise | Likely  Likely  Likely  Likely  Likely  Likely  Likely | Very low  Very low  Very low  Very low  Very low  Very low  Very low |
| **Key**: Pain Coping Inventory (PCI (strategy number used); Body Mass Index (BMI); Musculoskeletal (MSK); Passive Range of Movement (PROM); Western Ontario and McMaster Universities Osteoarthritis Index (WOMAC); Regression Coefficient (RC); Risk Ratio (RR); Odds Ratio (OR); Confidence Interval (CI).  RC classification: Value >0 = greater reduction in pai/improved function; <0 = less reduction in pain or functioning  RC classification of effect size: Small if measures between -1.4 to 0 and 0 to 1.4, moderate -1.41 to -3.4 and 1.4 to 3.4, large > -3.41 and >3.41  OR/HR/RR Classification of effect size: Small if measures between 0.66-1 and 1 to 2.4, moderate 0.33-0.65 and 2.41 to 4.4, large <0.32 and >4.41.  Effect size and confidence intervals in **bold** text indicate prognostic value (>moderate effect size with or without narrow CI’s or small effect size with narrow CI’s) | | | | | | | | | | | | | | | |
