# Supplementary material for: Prognostic factors associated with changes in knee pain outcomes, identified from initial primary care consultation data. A systematic literature review
Source: Ann Med. 2023 Jan 27;55(1):401–18. doi: 10.1080/07853890.2023.2165706 (PMC9888457; doi:10.1080/07853890.2023.2165706)
Supplement: Supplemental Material [file IANN_A_2165706_SM7575.docx]

**Supplementary file 9: Reasons for exclusion of articles in data synthesis**

|  | Reference | Exclusion reason |
| --- | --- | --- |
|  | (Ahmadi, Mousavi, Saffarzadeh, Hajiesmaeili, & Habibipour, 2020) | No knee outcomes investigated |
|  | (Ayaz, Panchbhavi, Kashif, Abdullah, & Akhtar, 2020) | No knee outcomes investigated |
|  | (Bacca, Celestino, Barela, Lima, & Barela, 2020) | No knee outcomes investigated |
|  | (Banerjee, Burkholder, Sana, & Szirony, 2020) | No knee outcomes investigated |
|  | (Barochiner & Martínez, 2020) | No knee outcomes investigated |
|  | (Bas, Senturk, Burnaz, Timur, & Kalkan, 2021) | No knee outcomes investigated |
|  | (Campbell, et al., 2021) | No knee outcomes investigated |
|  | (Chassagnon & Paragios, 2020) | No knee outcomes investigated |
|  | (Chen, et al., 2020) | No knee outcomes investigated |
|  | (Chong, Kashyap, Schauer, O'Rourke, & Husni, 2014) | Not primary care |
|  | (Cottrell, Roddy, Rathod, Porcheret, & Foster, 2016) | No knee outcomes investigated |
|  | (Dybowski, 2020) | No knee outcomes investigated |
|  | (Džiugys, Bieliūnas, Skarbalius, Misiulis, & Navakas, 2020) | No knee outcomes investigated |
|  | (Ektas, et al., 2020) | Not primary care |
|  | (Elangovan, et al., 2020) | No knee outcomes investigated |
|  | (Eshraghi, Khan, Said, Velasco, & Guirguis, 2020) | Not primary care |
|  | (Eaton, et al., 2012) | Conference abstract |
|  | (Fakhfakh, Bouaziz, Gargouri, & Chaari, 2020) | No knee outcomes investigated |
|  | (Fan, et al., 2020) | No knee outcomes investigated |
|  | (Fatima, et al., 2020) | Undergone surgery |
|  | (Feria-Romero, et al., 2020) | No knee outcomes investigated |
|  | (Fernandes, et al., 2020) | No knee outcomes investigated |
|  | (Flisiak, et al., 2021) | No knee outcomes investigated |
|  | (S. Fu, et al., 2020) | No knee outcomes investigated |
|  | (Y.-q. Fu, et al., 2020) | No knee outcomes investigated |
|  | (Gande & Irrgang, 2012) | Conference abstract |
|  | (Gijon, Metheringham, Paston, & Durrant, 2020) | No knee outcomes investigated |
|  | (Glaviano, Boling, & Fraser, 2020) | Not primary care |
|  | (Grandal, et al., 2020) | No knee outcomes investigated |
|  | (Hamilton, et al., 2020) | Undergone surgery |
|  | (Hao, Lv, et al., 2020; Hao, Zhang, et al., 2020) | No knee outcomes investigated |
|  | (Harrison-Brown, et al., 2020) | No knee outcomes investigated |
|  | (Hernández, Urrea, & Bascoy, 2020) | No knee outcomes investigated |
|  | (Helminen, Arokoski, Selander, & Sinikallio, 2020) | No primary care |
|  | (Hua, et al., 2019) | No knee outcomes investigated |
|  | (Husted, Bandholm, Rathleff, Troelsen, & Kirk, 2020) | No knee outcomes investigated |
|  | (Iglesias-Osores, et al., 2021) | No knee outcomes investigated |
|  | (Iwabuchi, et al., 2019) | Not primary care |
|  | (Iversen, et al., 2018) | Not primary care |
|  | (J, Lonnqvist, Nielsen, S, & Jensen, 2011) | Undergone surgery |
|  | (Jegatheesan, Scholes, Tuckerman, & Bell, 2020) | Undergone surgery |
|  | (S. Jiang, Zanazzi, & Hassanpour, 2021; W. Jiang, Dong, Hu, Wan, & Wang, 2020) | No knee outcomes investigated |
|  | (Jinks, et al., 2011) | No knee outcomes investigated |
|  | (Kharaz, Fang, Welting, Peffers, & Comerford, 2020) | Not primary care |
|  | (Kurozumi, et al., 2020) | No knee outcomes investigated |
|  | (Lagunas-Rangel & Chávez-Valencia, 2020) | No knee outcomes investigated |
|  | (Laing, et al., 2020) | No knee outcomes investigated |
|  | (Landsmeer, et al., 2019) | No knee pain at baseline |
|  | (Lazarus, Audrey, Wangsaputra, Tamara, & Tahapary, 2020) | No knee outcomes investigated |
|  | (Li, et al., 2020) | No knee outcomes investigated |
|  | (Lipunova, Bryan, & Zeegers, 2020) | No knee outcomes investigated |
|  | (Liu, et al., 2020) | No knee outcomes investigated |
|  | (Lu, Kong, Li, Jiang, & Tang, 2020) | No knee outcomes investigated |
|  | (Magu, Aggarwal, Behera, & Khurana, 2020) | Not primary care |
|  | (Marchetti, et al., 2020) | Not primary care |
|  | (Mark-Christensen, Juhl, Thorborg, & Bandholm, 2020) | Not primary care |
|  | (Martín-Aguilar, et al., 2020) | No knee outcomes investigated |
|  | (Moradi, et al., 2019) | Non-human |
|  | (M, C, & K P, 2008) | Not primary care |
|  | (Naing, et al., 2020) | No knee outcomes investigated |
|  | (Nazir, Ali, & Akhtar, 2020) | Not primary care |
|  | (O’Sullivan & Wright, 2020) | No knee outcomes investigated |
|  | (Okamura, et al., 2020) | No knee outcomes investigated |
|  | (Ogunbode, Adebusoye, Olowookere, & Alonge, 2014) | Cross sectional |
|  | (Parzefall, et al., 2020) | Not primary care |
|  | (Peat, et al., 2006) | Not primary care |
|  | (Peng, et al., 2020) | No knee outcomes investigated |
|  | (Poirion, Chaudhary, Huang, & Garmire, 2020) | No knee outcomes investigated |
|  | (Rhon, Hando, & Deyle, 2016) | Not primary care |
|  | (Richette, et al., 2009) | No knee outcomes investigated |
|  | (Roelker, et al., 2020) | Not primary care |
|  | (Roth, Howell, & Hull, 2019) | Not primary care |
|  | (Ruiz-Irastorza, et al., 2020) | No knee outcomes investigated |
|  | (Salas, Pulido, Montoya, & Ruiz, 2020) | No knee outcomes investigated |
|  | (Schiphof, Waarsing, Oei, & Bierma-Zeinstra, 2016) | Conference abstract |
|  | (Schmidt, et al., 2020) | No knee outcomes investigated |
|  | (Sigurðsson & Briem, 2019) | Not primary care |
|  | (Simon, et al., 2020) | No knee outcomes investigated |
|  | (Singh, et al., 2020) | No knee outcomes investigated |
|  | (Siston, et al., 2020) | Undergone surgery |
|  | (Sousa, et al., 2020) | No knee outcomes investigated |
|  | (Staines, Hardy, Samvelyan, Ward, & Cooper, 2020) | Not primary care |
|  | (Stewart, et al., 2020) | No knee outcomes investigated |
|  | (Tan, et al., 2020) | No knee outcomes investigated |
|  | (Tang, et al., 2020) | No knee outcomes investigated |
|  | (Thompson, Ahmed, Weldon, Efthimiou, & Stubbs, 2020) | Not primary care |
|  | (Tighe, et al., 2020) | No knee outcomes investigated |
|  | (Uhlrich, et al., 2020) | Not primary care |
|  | (van Dam, et al., 2020) | No knee outcomes investigated |
|  | (Verges Milano, et al., 2016) | Not primary care |
|  | (Vrabie, Gangal, & Gangal, 2020) | No knee outcomes investigated |
|  | (Wang, Singh, & Antony, 2020) | Not primary care |
|  | (Warren & Birol, 2020) | No knee outcomes investigated |
|  | (Williams, et al., 2020) | Undergone surgery |
|  | (Wright, Katz, Abrams, Solomon, & Losina, 2013) | No knee outcomes investigated |
|  | (Wynants, et al., 2020) | Not primary care |
|  | (Zeng, et al., 2020; Zhan, et al., 2020; J. Zhang, et al., 2020; Yan Zhang, et al., 2020; Y. Zhang, Zhang, & Hu, 2020; Z. Zhang, Yang, & Zhang, 2020) | No knee outcomes investigated |
