# Supplementary material for: Prognostic factors associated with changes in knee pain outcomes, identified from initial primary care consultation data. A systematic literature review
Source: Ann Med. 2023 Jan 27;55(1):401–18. doi: 10.1080/07853890.2023.2165706 (PMC9888457; doi:10.1080/07853890.2023.2165706)
Supplement: Supplemental Material [file IANN_A_2165706_SM7285.docx]

**Supplementary file 8: Appraisal of Quality of Evidence; GRADE Evaluation**

|  |  |  | | | | **Univariate** | **Multivariate** | **GRADE factors** | | | | | | | | |
| --- | --- | --- | --- | --- | --- | --- | --- | --- | --- | --- | --- | --- | --- | --- | --- | --- |
| **Outcome** | **Studies** | **Potential prognostic factors identified** | **Number of participants** | **Number of studies** | **Number of Cohorts** | **+ 0 -** | **+ 0 -** | **Phase** | **Study limitations** | **Inconsistency** | **Indirectness** | **Imprecisions** | **Publication bias** | **Moderate/large effect size** | **Dose effect** | **Overall quality** |
| Pain | **Three-month follow up** | | | | | | | | | | | | | | | |
|  | Van Der Waa (30) | Sex | 251 | 1 | 1 | ? | _-_ | 1 | X | X | ✓ | ✓ | X | X | X | + |
|  | Van Der Waal (30) | BMI >30 | 251 | 1 | 1 | ? | + | 1 | X | X | ✓ | ✓ | X | X | X | + |
|  | Van Der Waal (30) | Duration of the knee complaint | 251 | 1 | 1 | ? | - | 1 | X | X | ✓ | ✓ | X | X | X | + |
|  | Van Der Waal (30) | Cause overload during unusual activities | 251 | 1 | 1 | ? | - | 1 | X | X | ✓ | ✓ | X | X | X | + |
|  | Van Der Waal (30) | Baseline pain | 251 | 1 | 1 | ? | + | 1 | X | X | ✓ | ✓ | X | X | X | + |
|  | Van Der Waal (30) | PCI Distress Middle Vs. low | 251 | 1 | 1 | ? | - | 1 | X | X | ✓ | ✓ | X | ✓ | X | ++ |
|  | Van Der Waal (30) | Coexisting MSK complaints | 251 | 1 | 1 | ? | - | 1 | X | X | ✓ | ✓ | X | X | X | + |
|  | Van Der Waal (30) | ACSM position stand recommendations | 251 | 1 | 1 | ? | + | 1 | X | X | ✓ | ✓ | X | X | X | + |
|  | Van Der Waal (30) | Cause, overload during usual activities | 251 | 1 | 1 | ? | 0 | 1 | X | X | ✓ | ✓ | X | X | X | + |
|  | Van Der Waal (30) | Distress-middle vs lowest tertitle | 251 | 1 | 1 | ? | 0 | 1 | X | X | ✓ | ✓ | X | X | X | + |
|  | **12-month follow up** | | | | | | | | | | | | | | | |
|  | Van Der Waal (30) | Non-traumatic knee complaint history | 251 | 1 | 1 | ? | - | 1 | X | X | ✓ | ✓ | X | X | X | + |
|  | Van Der Waal (30) | Baseline pain present | 251 | 1 | 1 | ? | + | 1 | X | X | ✓ | ✓ | X | X | X | + |
|  | Van Der Waal (30) | PCI distraction high Vs. low | 251 | 1 | 1 | ? | - | 1 | X | X | ✓ | ✓ | X | X | X | + |
|  | Van Der Waal (30) | PCI distress high Vs. low | 251 | 1 | 1 | ? | - | 1 | X | X | ✓ | ✓ | X | ✓ | X | ++ |
|  | Van Der Waal (30) | Vitality | 251 | 1 | 1 | ? | + | 1 | X | X | ✓ | ✓ | X | X | X | + |
|  | Van Der Waal (30) | Cause, injury during exercise | 251 | 1 | 1 | ? | 0 | 1 | X | X | ✓ | ✓ | X | X | X | + |
|  | Van Der Waal (30) | PCI distraction mid Vs. low | 251 | 1 | 1 | ? | 0 | 1 | X | X | ✓ | ✓ | X | X | X | + |
|  | Van Der Waal (30) | PCI distress mid Vs. low | 251 | 1 | 1 | ? | 0 | 1 | X | X | ✓ | ✓ | X | X | X | + |
|  | **54-month follow up** | | | | | | | | | | | | | | | |
|  | Bastick et al (23) | Higher BMI | 705 | 1 | 1 | ? | ? | 1 | X | X | ✓ | ✓ | X | X | X | + |
|  | Bastick et al (23) | Lower level of education | 705 | 1 | 1 | ? | ? | 1 | X | X | ✓ | ✓ | X | ✓ | X | ++ |
|  | Bastick et al (23) | Greater Comorbidity | 705 | 1 | 1 | ? | ? | 1 | X | X | ✓ | ✓ | X | ✓ | X | ++ |
|  | Bastick et al (23) | Higher activity limitation scores | 705 | 1 | 1 | ? | ? | 1 | X | X | ✓ | ✓ | X | X | X | + |
|  | Bastick et al (23) | Joint space tenderness | 705 | 1 | 1 | ? | ? | 1 | X | X | ✓ | ✓ | X | x | x | + |
| Persisting knee symptoms | Belo et al (24) | Age >60 years | 480 | 1 | 1 | ? | ? | 1 | X | X | ✓ | X | X | X | X | + |
|  | Belo et al (24); Kastelein et al (26) | Education level | 652 | 2 | 2 | ? | ? and + | 1 | X | ✓, | ✓ | X | X | ✓ | X | ++ |
|  | Belo et al (24) | Kinesiophobia | 480 | 1 | 1 | ? | ? | 1 | X | X | ✓ | X | X | X | X | + |
|  | Belo et al (24) | Comorbidity of MSK system | 480 | 1 | 1 | ? | ? | 1 | X | X | ✓ | X | X | X | X | + |
|  | Belo et al (24) | Non-traumatic knee history symptoms | 480 | 1 | 1 | ? | ? | 1 | X | X | ✓ | X | X | ✓ | X | + |
|  | Belo et al (26); Kastelein et al (26) | Bilateral symptoms | 652 | 2 | 2 | ? | ? and + | 1 | X | ✓, | ✓ | x | X | ✓ | X | ++ |
|  | Belo et al (24) | >3-month symptom duration | 480 | 1 | 1 | ? | ? | 1 | X | X | ✓ | X | X | X | X | + |
|  | Belo et al (24) | Crepitus of PROM extension | 480 | 1 | 1 | ? | ? | 1 | X | X | ✓ | X | X | X | X | + |
|  | Kastelein et al (26) | BMI >25 | 172 | 1 | 1 | + | 0 | 1 | X | X | ✓ | X | X | X | X | + |
|  | Kastelein et al (26) | Self-reported crepitus knee | 172 | 1 | 1 | 0 | 0 | 1 | X | X | ✓ | X | X | X | X | + |
|  | Belo et al (26) | History of traumatic knee symptoms | 480 | 1 | 1 | ? | ? | 1 | X | X | ✓ | X | X | x | X | + |
| Unfavourable outcome | Kastelein et al (25) | Low/Middle education level | 549 | 1 | 1 | + | + | 1 | X | X | ✓ | ✓ | X | X | X | + |
|  | Kastelein et al (25) | Comorbidity skeletal system | 549 | 1 | 1 | + | + | 1 | X | X | ✓ | ✓ | X | X | X | + |
|  | Kastelein et al (25) | Poor mental Health (SF-36 score <50) | 549 | 1 | 1 | + | + | 1 | X | X | ✓ | ✓ | X | ✓ | X | ++ |
|  | Kastelein et al (25) | >3-month symptom duration | 549 | 1 | 1 | + | + | 1 | X | X | ✓ | ✓ | X | X | X | + |
|  | Kastelein et al (25) | Bilateral knee symptoms | 549 | 1 | 1 | + | + | 1 | X | X | ✓ | ✓ | X | X | X | + |
|  | Kastelein et al (25) | Self-report warm knee | 549 | 1 | 1 | + | + | 1 | X | X | ✓ | ✓ | X | X | X | + |
|  | Kastelein et al (25) | History of nontraumatic knee symptoms | 549 | 1 | 1 | + | + | 1 | X | X | ✓ | ✓ | X | ✓ | X | ++ |
|  | Kastelein et al (25) | Valgus | 549 | 1 | 1 | + | + | 1 | X | X | ✓ | ✓ | X | X | X | + |
|  | Kastelein et al (25) | Pain passive flexion | 549 | 1 | 1 | + | + | 1 | X | X | ✓ | ✓ | X | X | X | + |
|  | Kastelein et al (25) | Pain passive extension | 549 | 1 | 1 | + | + | 1 | X | X | ✓ | ✓ | X | X | X | + |
|  | Kastelein et al (25) | Bony enlargement of joint | 549 | 1 | 1 | + | + | 1 | X | X | ✓ | ✓ | X | ✓ | X | ++ |
|  | Kastelein et al (25) | Limitation during daily function (WOMAC >20) | 549 | 1 | 1 | + | 0 | 1 | X | X | ✓ | ✓ | X | X | X | + |
|  | Kastelein et al (25) | Comorbidity skeletal system | 549 | 1 | 1 | + | 0 | 1 | X | X | ✓ | ✓ | X | x | x | + |
| Self-reported perceived recovery | Kastelein et al (27) | Age | 328 | 1 | 1 | + | + | 1 | X | X | ✓ | X | X | X | X | + |
|  | Kastelein et al (27) | Poor general health (SF 36<50) | 328 | 1 | 1 | + | + | 1 | X | X | ✓ | X | X | ✓ | X | + |
|  | Kastelein et al (27) | History of non-traumatic knee symptoms | 328 | 1 | 1 | + | + | 1 | X | X | ✓ | X | X | X | X | + |
|  | Kastelein et al (27) | Floating patella | 328 | 1 | 1 | + | + | 1 | X | X | ✓ | X | X | ✓ | X | + |
|  | Kastelein et al (27) | Age | 328 | 1 | 1 | + | + | 1 | X | X | ✓ | X | X | X | X | + |
|  | Kastelein et al (27) | BMI | 328 | 1 | 1 | + | + | 1 | X | X | ✓ | X | X | ✓ | X | + |
|  | Kastelein et al (27) (27) | non MSK comorbidity | 328 | 1 | 1 | + | + | 1 | X | X | ✓ | X | X | X | X | + |
|  | Kastelein et al | Self-reported crepitus | 328 | 1 | 1 | + | + | 1 | X | X | ✓ | X | X | X | X | + |
|  | Kastelein et al (27); Van Der Waal (30) | History of non-traumatic knee symptoms | 579 | 2 | 2 | + + | + + | 1 | X | ✓, | ✓ | X | X | X | X | + |
|  | Kastelein et al (27) | Anterior drawer test laxity | 328 | 1 | 1 | + | 0 | 1 | X | X | ✓ | X | X | X | X | + |
|  | Kastelein et al (27) | Effusion popliteal fossa | 328 | 1 | 1 | 0 | 0 | 1 | X | X | ✓ | X | X | x | X | + |
| Poor functional outcome | **Three-month follow up** | | | | | | | | | | | | | | | |
|  | Van der Waal et al (30) | Age | 251 | 1 | 1 | - | - | 1 | X | X | ✓ | ✓ | X | X | X | + |
|  | Van der Waal et al (30) | Female | 251 | 1 | 1 | ? | - | 1 | X | X | ✓ | ✓ | X | X | X | + |
|  | Van der Waal et al (30) | Duration of knee complaint | 251 | 1 | 1 | - | - | 1 | X | X | ✓ | ✓ | X | ✓ | X | ++ |
|  | Van der Waal et al (30) | WOMAC pain | 251 | 1 | 1 | + | - | 1 | X | X | ✓ | ✓ | X | X | X | + |
|  | Van der Waal et al (30) | WOMAC functioning | 251 | 1 | 1 | + | + | 1 | X | X | ✓ | ✓ | X | X | X | + |
|  | Van der Waal et al (30) | PCI sub-scale (3); distress high Vs lowest tertitle | 251 | 1 | 1 | - | - | 1 | X | X | ✓ | ✓ | X | ✓ | X | ++ |
|  | Van der Waal et al (30) | Complaints of upper and lower extremity Vs knee only complaint | 251 | 1 | 1 | - | - | 1 | X | x | ✓ | ✓ | x | ✓ | X | ++ |
|  | **12-month follow up** | | | | | | | | | | | | | | | |
|  | Van der Waal et al (30) | Age | 251 | 1 | 1 | - | - | 1 | X | X | ✓ | ✓ | X | X | X | + |
|  | Van der Waal et al (30) | Duration of knee complaint | 251 | 1 | 1 | - | - | 1 | X | X | ✓ | ✓ | X | ✓ | X | ++ |
|  | Van der Waal et al (30) | WOMAC stiffness | 251 | 1 | 1 | + | - | 1 | X | X | ✓ | ✓ | X | X | X | + |
|  | Van der Waal et al (30) | WOMAC functioning | 251 | 1 | 1 | + | + | 1 | X | X | ✓ | ✓ | X | X | X | + |
|  | Van der Waal et al (30) | PCI sub-scale (4); retreating mid Vs. low | 251 | 1 | 1 | + | + | 1 | X | X | ✓ | ✓ | X | ✓ | X | ++ |
|  | Van der Waal et al (30) | PCI sub-scale (2); distraction high Vs. low | 251 | 1 | 1 | - | - | 1 | X | X | ✓ | ✓ | X | ✓ | X | ++ |
|  | Van der Waal et al (30) | PCI sub scale (3) reducing demands mid Vs. low | 251 | 1 | 1 | + | - | 1 | X | X | ✓ | ✓ | X | ✓ | X | ++ |
|  | Van der Waal et al (30) | PCI sub scale (3) reducing demands high Vs. low | 251 | 1 | 1 | + | - | 1 | X | X | ✓ | ✓ | X | X | X | + |
|  | Van der Waal et al (30) | PCI sub scale 5) distress mid Vs. low | 251 | 1 | 1 | + | - | 1 | X | X | ✓ | ✓ | X | X | X | + |
|  | Van der Waal et al (30) | Coexisting complaint lower extremity Vs knee only complaint | 251 | 1 | 1 | + | - | 1 | X | X | ✓ | ✓ | X | ✓ | X | ++ |
|  | Van der Waal et al (30) | Meeting ACSM position stand Vs not meeting norm | 251 | 1 | 1 | + | - | 1 | X | X | ✓ | ✓ | X | ✓ | X | ++ |
|  | Van der Waal et al (30) | Social support | 251 | 1 | 1 | + | - | 1 | X | X | ✓ | ✓ | X | X | X | + |
|  | Van der Waal et al (30) | PCI sub-scale (4); retreating high Vs. low | 251 | 1 | 1 |  | 0 | 1 | X | X | ✓ | ✓ | X | ✓ | x | ++ |
|  | Van der Waal et al (30) | PCI sub-scale (2); distraction mid Vs. low | 251 | 1 | 1 |  | 0 | 1 | X | X | ✓ | ✓ | X | ✓ | x | ++ |
|  | **18-month follow up** | | | | | | | | | | | | | | | |
|  | Mallen et al (28) | Age 60-69 | 621 | 1 | 1 | + | + | 1 | X | X | ✓ | ✓ | X | X | X | + |
|  | Mallen et al (28) | Age 70+ | 621 | 1 | 1 | + | + | 1 | X | X | ✓ | ✓ | X | X | X | + |
|  | Mallen et al (28) | BMI 25-29.9 | 621 | 1 | 1 | + | + | 1 | X | X | ✓ | ✓ | X | X | X | + |
|  | Mallen et al (28) | BMI >30 | 621 | 1 | 1 | + | + | 1 | X | X | ✓ | ✓ | X | X | X | + |
|  | Mallen et al (28) | Possible anxiety | 621 | 1 | 1 | + | + | 1 | X | X | ✓ | ✓ | X | X | X | + |
|  | Mallen et al (28) | Probable anxiety | 621 | 1 | 1 | + | + | 1 | X | X | ✓ | ✓ | X | X | X | + |
|  | Mallen et al (28) | Chronic pain grade II | 621 | 1 | 1 | + | + | 1 | X | X | ✓ | ✓ | X | X | X | + |
|  | Mallen et al (28) | Chronic pain grade III | 621 | 1 | 1 | + | + | 1 | X | X | ✓ | ✓ | X | X | X | + |
|  | Mallen et al (28) | Chronic pain grade IV | 621 | 1 | 1 | + | 0 | 1 | X | X | ✓ | ✓ | X | X | X | + |
|  | Thomas et al (29) | Duration of morning stiffness <30 mins | 621 | 1 | 1 | + | + | 1 | X | X | ✓ | X | X | X | X | + |
|  | Thomas et al (29) | Local tender point count 2 | 621 | 1 | 1 | + | + | 1 | X | X | ✓ | X | X | X | X | + |
|  | Thomas et al (29) | Local tender point count 3 | 621 | 1 | 1 | + | + | 1 | X | X | ✓ | X | X | X | X | + |
|  | Thomas et al (29) | Local tender point count 4-6 | 621 | 1 | 1 | + | + | 1 | X | X | ✓ | X | X | X | X | + |
|  | Thomas et al (29) | Single leg stand 10-29 seconds | 621 | 1 | 1 | + | + | 1 | X | X | ✓ | X | X | X | X | + |
|  | Thomas et al (29) | Single leg stand 4-9 seconds | 621 | 1 | 1 | + | + | 1 | X | X | ✓ | X | X | X | X | + |
|  | Thomas et al (29) | Single leg stand <4 seconds | 621 | 1 | 1 | + | + | 1 | X | X | ✓ | X | X | X | X | + |
|  | Thomas et al (29) | Bilateral Knee pain present | 621 | 1 | 1 | + | 0 | 1 | X | X | ✓ | X | X | X | X | + |
|  | Thomas et al (29) | Inactivity gelling present | 621 | 1 | 1 | + | 0 | 1 | X | X | ✓ | X | X | X | X | + |
|  | Thomas et al (29) | Duration of morning stiffness >30 min | 621 | 1 | 1 | 0 | 0 | 1 | X | X | ✓ | X | X | X | X | + |
|  | Thomas et al (29) | Local tender point count 1 | 621 | 1 | 1 | 0 | 0 | 1 | X | X | ✓ | X | X | X | x | + |
| Phase, phase of investigation. For univariate and multivariate analyses: +, significant effect with a positive value; 0, non-significant effect; -, significant effect with a negative value; ?. effect size not provided. For GRADE factors: ✓, no serious limitations; ✕, serious limitations (or not present for moderate/large effect size, dose effect); unclear, unable to rate item based on available information. For overall quality of evidence: +, very low; ++, low; +++, moderate; ++++, high | | | | | | | | | | | | | | | | |
