# Supplementary material for: Prognostic factors associated with changes in knee pain outcomes, identified from initial primary care consultation data. A systematic literature review
Source: Ann Med. 2023 Jan 27;55(1):401–18. doi: 10.1080/07853890.2023.2165706 (PMC9888457; doi:10.1080/07853890.2023.2165706)
Supplement: Supplemental Material [file IANN_A_2165706_SM7163.docx]

**Supplementary file 5: Checklist for Critical Appraisal and Data Extraction for Systematic Reviews of Prediction Modelling Studies - Prognostic Factors**

| **PAPER:** |
| --- |
| **Source of data:** |
| Source of data (eg, cohort, case control, randomised trial, or registry data) |
| **Participants** |
| Participant eligibility and recruitment method (eg, consecutive participants, location, number of centres, setting, inclusion and exclusion criteria) |
| Participant description |
| Details of treatments received (if relevant) |
| Study dates |
| **Outcomes to be predicted:** |
| Definition and method for measurement of outcomes |
| Was the same outcome definition (and method for measurement) used in all participants? |
| Types of outcomes (eg, single or combined endpoints)? |
| Were the outcomes assessed without knowledge of the candidate prognostic factors (that is, blinded)? |
| Were candidate prognostic factors part of the outcome (eg, when using a panel or consensus outcome measurement)? |
| Time of outcome occurrence or summary of duration of follow up |
| **Outcomes to be predicted: (index and comparator prognostic factors):** |
| Number and type of prognostic factors (eg, obtained from demographics, patient history, physical examination, additional testing, disease characteristics) |
| Definition and method for measurement of prognostic factors |
| Timing of prognostic factor measurement (eg, at patient presentation, diagnosis, treatment initiation, at the end of surgery) |
| Were prognostic factors assessed blinded for outcome, and for each other (if relevant)? |
| Handling of prognostic factors in the analysis (eg, continuous, linear, non-linear transformations or categorised) |
| **Sample Size:** |
| Was a sample size calculation conducted and, if so, how? |
| Number of participants and number of outcomes or events |
| Number of outcomes or events in relation to the number of candidate prognostic factors (events per variable) |
| **Missing data:** |
| Number of participants with any missing value (in the prognostic factors and outcomes) |
| Number of participants with missing data for each prognostic factor of interest |
| Details of attrition (loss to follow up) and, for time-to-event outcomes, number of censored observations (ideally in each category for those categorical prognostic factors of interest) |
| Handling of missing data (eg, complete case analysis, imputation, or other methods) |
| **Analysis** |
| Modelling method (eg, linear, logistic, Cox, parametric survival, competing risks) regression) |
| How modelling assumptions were checked; in particular, for time-to-event outcomes and the analysis of hazard ratios, the method for assessing non-proportional hazards (non-constant hazard ratios over time) |
| Method for selection of prognostic factors for inclusion in multivariable modelling (eg, all candidate prognostic factors considered, preselection of established prognostic factors, retain only those significant from univariable analysis) |
| Method for selection or exclusion of prognostic factors (including those of interest and those used as adjustment factors) during multivariable modelling (eg, backward or forward selection, or full model approach including all factors regardless), and criteria used for any selection or exclusion (eg, P value, Akaike information criterion) |
| Method of handling each continuous prognostic factor (eg, dichotomisation, categorisation, linear, non-linear), including values of any cut points used and their justification; for non-linear trends, the method of identifying non-linear relationships (eg, splines, fractional polynomials) |
| **Results** |
| Unadjusted and adjusted prognostic effect estimates (eg, risk ratios, odds ratios, hazard ratios, mean differences) for each prognostic factor of interest, and the corresponding 95% confidence interval (or variance or standard error). Details of any non-linear relationships and whether modelling assumptions hold; in particular, for time-to-event outcomes, any evidence of non-proportional hazards (non-constant hazard ratios) for each prognostic factor of interest |
| For each extracted adjusted prognostic effect estimate of interest, the set of adjustment factors used |
| **Interpretation and discussion:** |
| Interpretation of presented results |
| Comparison with other studies, discussion of generalisability, strengths and limitations |
