# Supplementary material for: Prognostic factors associated with changes in knee pain outcomes, identified from initial primary care consultation data. A systematic literature review
Source: Ann Med. 2023 Jan 27;55(1):401–18. doi: 10.1080/07853890.2023.2165706 (PMC9888457; doi:10.1080/07853890.2023.2165706)
Supplement: Supplemental Material [file IANN_A_2165706_SM7161.docx]

**Supplementary file 4: CINAHL Search String**

***Condition***

1. **Text words:** " Knee joint” or knee or patella or “patell?femoral$” or “tibi?femoral$”
2. **Text words:** Pain or chronic pain or ache or arthralgia or discomfort or soreness
3. **Text words:** Sprains or strains or " knee osteoarthritis” or knee injuries or ligaments or muscles or tendons or "Patell?femoral pain$" or menis* or burs*
4. **1 and 2 and 3**
5. ***Prognostic factors***
6. **Text words: "** Diagnostic Imaging” or " Magnetic Resonance Imaging" or "Blood Culture" or " Physical Examination" or " Psychosocial Functioning" or " Signs and Symptoms" or **“**giving way" or instability or crepitus or medication or corticosteroid$ or Injection or Exercise*
7. ***Setting***
8. **Text words: "**Primary Health Care” or Physicians, or " Family Practice" or “general practice” or “family physician” or “family doctor” or GP or "primary care clinician$" or “physiotherapist$” or “first contact practitioner$” or “nurse practitioner$” or “physicians associate$”
9. ***Publication Type***
10. **Text words:** Prognosis or " statistical models” or " Epidemiologic Studies" or " Cohort Studies" or " Multivariate Analysis" or Probability or " Proportional Hazard Model" or predict or course or "risk factor" or "causal factor" or “randomi?ed control” or “case control” or “logistic regression” or “machine learning” or “artificial intelligence”
11. ***Outcomes***
12. **Text words:** "Patient Reported Outcome Measures" or VAS or "visual analogue scale" or "numeric rating scale" or “general health” or health or radiographic or “kellgren-lawrence scale” or EQ5D or "western ontario and mcmaster universities osteoarthritis index" or “knee injury and osteoarthritis outcome score” or questionnaire
13. **4 and 5 and 6 and 7 and 8**
