# Supplementary material for: Prognostic factors associated with changes in knee pain outcomes, identified from initial primary care consultation data. A systematic literature review
Source: Ann Med. 2023 Jan 27;55(1):401–18. doi: 10.1080/07853890.2023.2165706 (PMC9888457; doi:10.1080/07853890.2023.2165706)
Supplement: Supplemental Material [file IANN_A_2165706_SM6506.docx]

**Supplementary file 2: EMBASE Search String**

***Condition***

1. **MeSH terms:** knee/ or patella/
2. **Text words:** patell?femoral$ or tibi?femoral$
3. **1 or 2**
4. **MeSH terms:** exp pain/ or chronic pain/
5. **Text words:** ache or arthralgia or discomfort or soreness
6. **4 or 5**
7. **MeSH terms:** sprain/ or muscle strain/ or knee osteoarthritis/ or knee injury/ or ligament/ or muscle/ or tendon/
8. **Text words:** patell?femoral pain$ or menis* or burs*
9. **7 or 8**
10. **3 and 6 and 9**

***Prognostic factors***

1. **MeSH terms:** diagnostic imaging/ or magnetic resonance imaging/ or blood culture/ or exp physical examination/ or exp social psychology/ or exp signs and symptoms/
2. **Text words:** giving way or instability or crepitus or medication or corticosteroid$ or injection or exercise*
3. **11 or 12**

***Setting***

1. **MeSH terms:** exp primary health care/ physicians/ or general practice/
2. **Text words:** family physician or family doctor or gp or primary care clinician$ or physiotherapist$ or first contact practitioner$ or nurse practitioner$ or physicians associate$
3. **14 or 15**

***Publication Type***

1. **MeSH terms:** prognosis/ exp statistical model/ or epidemiology/ or cohort analysis/ or multivariate analysis/ or probability/ or proportional hazards model/
2. **Text words:** predict or course or risk factor or causal factor or randomi?ed control or case control or logistic regression or machine learning or artificial intelligence
3. **17 or 18**

***Outcomes***

1. **MeSH terms:** exp patient-reported outcome/
2. **Text words:** vas or visual analogue scale or numeric rating scale or general health or health or radiographic or kellgren-lawrence scale or eq5d or western ontario and mcmaster universities osteoarthritis index or knee injury and osteoarthritis outcome score or questionnaire
3. **20 or 21**

**10 and 13 and 16 and 19 and 22**
