# Supplementary material for: Prognostic factors associated with changes in knee pain outcomes, identified from initial primary care consultation data. A systematic literature review
Source: Ann Med. 2023 Jan 27;55(1):401–18. doi: 10.1080/07853890.2023.2165706 (PMC9888457; doi:10.1080/07853890.2023.2165706)
Supplement: Supplemental Material [file IANN_A_2165706_SM6498.docx]

**Supplementary file 1: Medline Search String**

***Condition***

1. **MeSH terms:** Knee joint/ or knee/ or patella/
2. **Text words:** “patell?femoral$” or “tibi?femoral$”
3. **1 or 2**
4. **MeSH terms:** exp Pain/ or chronic pain/
5. **Text words:** ache or arthralgia or discomfort or soreness
6. **4 or 5**
7. **MeSH terms:** Sprains and strains/ or Osteoarthritis, knee/ or Knee injuries/ or ligaments/ or muscles/ or tendons/
8. **Text words:** "Patell?femoral pain$" or menis* or burs*
9. **7 or 8**
10. **3 and 6 and 9**

***Prognostic factors***

1. **MeSH terms:** Diagnostic Imaging/ or Magnetic Resonance Imaging/ or Blood Culture/ or exp Physical Examination/ or exp Psychosocial Functioning/ or exp Signs and Symptoms/
2. **Text words: “**giving way" or instability or crepitus or medication or corticosteroid$ or Injection or Exercise*
3. **11 or 12**

***Setting***

1. **MeSH terms:** exp Primary Health Care/ Physicians, Primary Care/ or Family Practice/
2. **Text words:** “general practice” or “family physician” or “family doctor” or GP or "primary care clinician$" or “physiotherapist$” or “first contact practitioner$” or “nurse practitioner$” or “physicians associate$”
3. **14 or 15**

***Publication Type***

1. **MeSH terms:** Prognosis/ exp Models, Statistical/ or Epidemiologic Studies/ or Cohort Studies/ or Multivariate Analysis/ or Probability/ or Proportional Hazard Model/
2. **Text words:** predict or course or "risk factor" or "causal factor" or “randomi?ed control” or “case control” or “logistic regression” or “machine learning” or “artificial intelligence”
3. **17 or 18**

***Outcomes***

1. **MeSH terms:** exp Patient Reported Outcome Measures/
2. **Text words:** VAS or "visual analogue scale" or "numeric rating scale" or “general health” or health or radiographic or “kellgren-lawrence scale” or EQ5D or "western ontario and mcmaster universities osteoarthritis index" or “knee injury and osteoarthritis outcome score” or questionnaire
3. **20 or 21**
4. **10 and 13 and 16 and 19 and 22**
